# Harmonizing existing climate change mitigation policy datasets with a hybrid machine learning approach

**DOI:** 10.1038/s41597-024-03411-z

**Published:** 2024-06-04

**Authors:** Libo Wu, Zhihao Huang, Xing Zhang, Yushi Wang

**Affiliations:** 1 School of Data Science, Fudan University, Shanghai, 200433 China; 2 Institute for Big Data, Fudan University, Shanghai, 200433 China; 3 School of Economics, Fudan University, Shanghai, 200433 China; 4 Shanghai Institute for Energy and Carbon Neutrality Strategy, Fudan University, Shanghai, 200433 China

**Keywords:** Climate-change policy, Climate-change mitigation

## Abstract

With the rapid proliferation of climate policies in both number and scope, there is an increasing demand for a global-level dataset that provides multi-indicator information on policy elements and their implementation contexts. To address this need, we developed the Global Climate Change Mitigation Policy Dataset (GCCMPD) using a semisupervised hybrid machine learning approach, drawing upon policy information from global, regional, and sector-specific sources. Differing from existing climate policy datasets, the GCCMPD covers a large range of policies, amounting to 73,625 policies of 216 entities. Through the integration of expert knowledge-based dictionary mapping, probability statistics methods, and advanced natural language processing technology, the GCCMPD offers detailed classification of multiple indicators and consistent information on sectoral policy instruments. This includes insights into objectives, target sectors, instruments, legal compulsion, administrative entities, etc. By aligning with the sector classification of the Intergovernmental Panel on Climate Change (IPCC) emission datasets, the GCCMPD serves to help policy-makers, researchers, and social organizations gain a deeper understanding of the similarities and distinctions among climate activities across countries, sectors, and entities.

## Background & Summary

With the first World Climate Conference in 1979 marking the start of an international focus on climate change issues, the number and scope of climate-related policies have increased substantially. At the global level, an international governance scheme formulated the Kyoto Protocol in 1997 and the Paris Agreement in 2015 to set up national mitigation targets and ancillary mechanisms. National, subnational and sectoral policies have also flourished in recent years, with some local consideration of other objectives such as environmental protection, economic development, equity thinking and sustainable development. Quantitative policy studies have emerged accordingly, either assessing the specific policy effect empirically or simulating the policy impacts virtually.

The literature focusing on the analysis of a single policy typically involves only a single country (or even a specific industry in a certain country)^1–6^ or a comparison of a few countries^7,8^, thus requiring a limited amount of policy data. A more detailed and consistent climate policy dataset enables large-scale quantitative analysis of global climate policies and provides new information for policy-makers. For example, through detailed classification based on direct and indirect laws, the differences in the coverage scope of greenhouse gas targets can be analysed^9^. Through the classification of instruments, various patterns of policy convergence and divergence across countries and the driving factors behind them can be compared^10^.

On the other hand, the differences in the circumstances of policy formulation and implementation are driving wide variations with similar policy instruments, which have not been fully captured for better evaluating the policy effects. In addition, some rising concerns, such as the constraints of policy adoption in developing economies, the synergistic effects of the policy mix, and the spillover effects of market-based instruments across regions, call for more comparative studies with global or cross-regional perspectives. Such demands in policy science require a more finely categorized, multi-indicator climate policy dataset that allows for convincing investigation of policy design under the premise of clarifying the implementation context.

Furthermore, the climate policy literature has diversified significantly with the emergence of new climate practices and the availability of more advanced data processing methods. According to SciVal (see Supplementary Information (SI1) for detailed search terms), the number of articles in climate policy-related research areas has been increasing annually since 1997, reaching 6,946 articles in 2022. Concurrently, the number of topics within the global climate policy research domain has also expanded, rising from 499 in 2018 to 751 in 2022 (Supplementary Figs. 1-2). Climate policy evolution represents one of the emerging branches within this research domain, encompassing various aspects such as policy coverage^9,11,12^, factors influencing policy adoption^10,13,14^, policy diffusion^15,16^, and policy themes and trends^17–21^. The utilization of policy quantity as the primary variable has broadened the scope of research on policy effects and their relationship with greenhouse gases^22–24^. At the same time, there has been a gradual shift towards more detailed and consistent exploration of policy design and comparison, adopting a mixed policy perspective^25–30^. In addition, research on the trade-offs between multiple goals^31–34^ also requires the support of multi-indicator policy datasets.

To the best of our knowledge, the existing global climate policy datasets are still not sufficient to meet the above research demands. The following points are taken as an example. (1) The lack of policy instruments for specific sectors prevents in-depth research on the policy mix for specific sectors. The lack of linkage between sectoral policies and sectoral carbon emissions, coupled with the varying focus of existing datasets on sectors^35^, further compounds these limitations. (2) Variations in policy versions and coverage can also undermine the robustness of research that relies on policy density as a metric^35^. (3) The separation of the law from a large number of supportive and weaker policies will also cause the role of supportive policies to be ignored^36^. The above shortcomings can be summarized as follows. First, the numbers and scopes of policies are still not complete, and updates cannot be made in time due to manual methods of data collection and processing. Second, there is a lack of consistent and more detailed policy categorization in comparable standards, partly due to the overlapping scope of sectors, complexity of entities involved, and difficulty in identifying indirect policies. These limitations also make it impractical to conduct comparative analysis for the identification of additional features across datasets. Additionally, studies comparing existing mainstream global datasets have shown that differences in data coverage and inconsistent classification standards lead to inconsistent results when using different datasets^35^. Finally, legal enforcement characteristics and information about administrative entities are crucial in driving changes in policy design. The aforementioned features may impact the practical outcomes of policy implementation and serve as a complement to policy ambitions^35,36^.

We attempted to construct the Global Climate Change Mitigation Policy Dataset (GCCMPD) to fill the gap between the existing climate policy databsets and the increasing demand for research by harmonizing the existing datasets with a hybrid machine learning approach. The GCCMPD currently includes 73,625 policies of 216 entities, with a substantial increase in the numbers and scopes of policies through a complete search of available sources. For each policy in the dataset, we extracted important policy features such as target sector, policy instrument, objective, binding force, executive/legislative, jurisdiction, etc. We mainly used semisupervised machine learning and combined various natural language processing methods to achieve consistent classification of each feature of the policy, which enhances the objectivity and extensibility of the data.

Compared with the existing datasets, our dataset can better respond to the demands of empirical studies and cross-national comparisons of policies. First, our dataset can be used to build more accurate policy indicators. On the one hand, our dataset includes more policy records than any individual dataset and has fewer omitted policies, which can ease the underestimation of the efforts of the entities to mitigate climate change. Coordinated climate policies can also enrich single or small n-case country case studies^30,35,37^. On the other hand, our dataset provides features of the policies, including the binding force, the executive/legislative and the objectives. Since the differences in the circumstances of policy formulation and implementation can vary greatly among policies with different binding forces (for example, laws and preparative instruments) and strongly influence the output of the policies, future studies can weigh the number of policies with their binding force or add the number of soft and hard laws into their model as two separate variables rather than simply counting the number of policies^23,24,35,36^. Second, our dataset enables us to measure the output of sectoral policies. Our dataset categorizes the policies into sectors according to the IPCC Sixth Assessment Report (AR6) standard and can be easily linked to emission datasets with the same standard, such as the Emissions Database for Global Atmospheric Research (EDGAR). Thus, with the sectoral emission data from these datasets and climate policy indicators calculated by sector instruments with our dataset, the sectoral emission reduction performance of the policy system (rather than the emission reduction performance of single policies^38–41^) can be measured. Third, the policy similarity information of our dataset supports a better exploration of climate policy diffusion. We provide information on the similarity of the context of climate policies, facilitating the inference of policy diffusion using both temporal and contextual data. This approach has the potential to outperform existing studies^42^, which primarily rely on inferring the probability of diffusion from one entity to another based solely on the time gap between the adoption of climate policies in the two entities. Finally, our dataset includes information on the binding force and policy objectives, enabling studies to explore the relationship between these features and other factors. For example, researchers can investigate how various conditions, such as economic or geographic factors, may influence the binding force of policies, as well as the differences in policy outcomes associated with different levels of binding force. Moreover, our dataset enables studies to focus on the dynamics between hard law and soft law, such as examining how soft law transitions into hard law and to what extent different types of laws influence emissions^43,44^.

## Methods

**Fig. 1:**
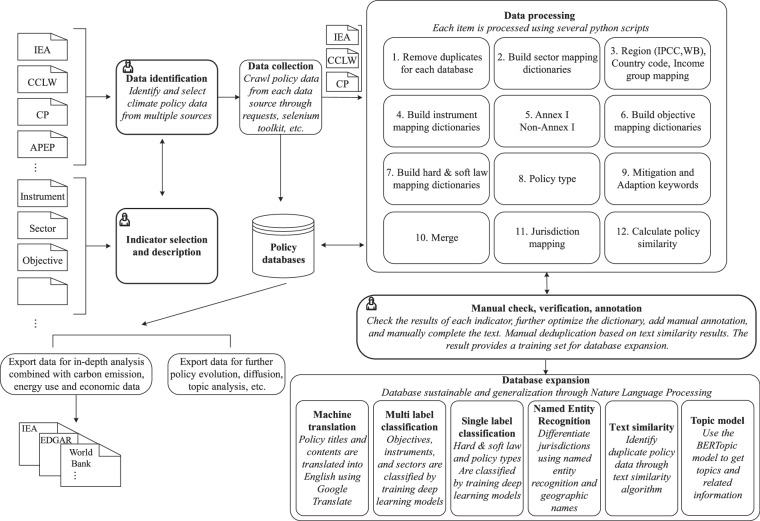
Overview of the framework for building the GCCMPD.

We constructed the GCCMPD using a multilevel process and framework (Fig. 1). Specifically, this entailed source identification and selection of policy data, selection of policy characteristic indicators, data collection, data processing, manual checks, verification, annotation, dataset expansion and some possible uses. The final data are also stored in different ways to facilitate various types of users.

### Data identification

Before identifying the data source, it is important to define the boundaries of the dataset described in this paper. At present, there is no clear definition of climate policy, and existing attempts to define climate policy have adopted very broad and vague scopes^20^. The reason is that climate policy covers a wide range of areas. For example, greenhouse gases and pollutants have common sources^45^, resulting in considerable overlap between climate policy and energy and environmental policy. Sometimes climate policy is directly defined as policies to reduce greenhouse gases and air pollution. Our purpose is to construct a detailed classification of multiple-indicator multipurpose datasets, so we use a relatively broad definition to define climate policy as a set of laws, regulations, strategies, and other measures related to climate change, which can be further divided into climate mitigation and climate adaptation policies. Dataset users can filter by attributes such as sources to obtain more targeted subsets of data, despite the broad boundaries of the dataset.

According to the above definition, the GCCMPD roughly consists of three sources (see Table 1). First, global datasets, the Climate Change Laws of the World (CCLW), the International Energy Agency (IEA) Policies and Measures Dataset, and the Climate Policy dataset (CP) were used. Second, regional datasets, such as the Asia Pacific Energy Portal (APEP) and the European Environment Agency (EEA), cover a certain area and have a considerable number of policies. The third category is datasets that focus on specialized fields such as technology, policy instruments, and legislation. These data are extremely specialized but narrow in scope. For the ECOLEX dataset, we selected climate-related subjects (see Supplementary Table 1 for details).

**Table 1:** Data sources for the GCCMPD.

Datasets	Records	Other description	Reference
**Global datasets**			
Climate Change Laws of the World (CCLW)	3,127	A national-level climate change legislation and policy dataset	https://climate-laws.org/
International Energy Agency (IEA) Policies and Measures Dataset	7,460	Global dataset covering climate, renewable energy, energy efficiency and many other fields	https://www.iea.org/policies/
Climate Policy dataset (CP)	5,782	A climate-related policy dataset from multiple data sources with varying levels of geographic and sectoral coverage	https://climatepolicydatabase.org/
**Regional datasets**			
Asia Pacific Energy Portal (APEP)	2,089	Focus on energy sector policy in the Asia-Pacific region	https://asiapacificenergy.org/
European Environment Agency (EEA)	2,304	Focus on climate change mitigation policies and measures in Europe	http://pam.apps.eea.europa.eu/
**Datasets focused on specialized domains such as technology, policy instruments, legislation**			
International Carbon Action Partnership (ICAP)	49	Focus on ETS policy in countries, subnational jurisdictions and supranational institutions around the world	https://icapcarbonaction.com/en/
Carbon Dioxide Removal (CDR NETs)	174	Focus on laws, proposals, reports and other policies on carbon dioxide removal (CDR) technologies or negative emission technologies (NET)	https://cdrlaw.org/technical-pathway/negative-emission-technologies/
Carbon Capture, Utilization and Storage (CDR CCUS)	189	Focus on carbon capture, utilization and storage (CCUS) technologies	https://cdrlaw.org/technical-pathway/carbon-capture-utilization-and-storage/
Climate Reregulation Tracker (CRT)	170	Focus on federal regulatory areas of climate mitigation and adaptation	https://climate.law.columbia.edu/content/climate-reregulation-tracker
ECOLEX Legislation	79,340	Focus on legislation in the field of environment and natural resource management	https://www.ecolex.org/result/?type = legislation
ECOLEX Treaty	810	Focus on bilateral or multilateral treaties in the field of environment and natural resource management	https://www.ecolex.org/result/?type = treaty

The selection methods for climate-related data in the ECOLEX dataset are provided in Supplementary Table 1.

We selected IEA, CP, and CCLW as the training sets for manual annotation, accounting for approximately 16% of all data sources (excluding the legal part of ECOLEX, which accounts for approximately 70%). Since the GCCMPD is a multi-indicator dataset, it is appropriate to choose authoritative and diverse data sources, and these three datasets have gradually been recognized by the academic community^18,22,23^. We first harmonized the three datasets to form a “core” dataset via the natural language processing technique and manual checking. The “core” dataset can support the demand of analysing global climate mitigation policies since these three datasets cover various types of climate policies (laws, commitment agreements, goals; national level, subnational level) from almost all of the entities in the world, which can basically guarantee the diversity and representativeness of the core dataset. Additionally, we noticed that the data coverage of the “core” dataset can be further improved by including data from other data sources. Then, we used the machine learning models trained on the manually checked core dataset to label data from other sources and formed a full dataset. The IEA, CP, and CCLW datasets have great potential to complement each other in terms of country coverage, sector coverage, laws and nonlaws, policy versions, policy scope, etc. CCLW is a specialized climate law dataset that collects climate laws or equally important policies^23^, with the highest policy importance but a relatively small number of policies. The IEA was the first of the three datasets to collect policy data on energy-related policies in the building sector. CP is a synthesized dataset containing policies from other datasets (including CCLW and IEA) and various reporting sources, characterized by detailed information on policy targets^9,12^, instruments and sectors^11^. After harmonizing with the IEA and CP datasets, the following complements were formed: (1) subnational policies and early policy versions; (2) nonlegal supportive “weaker” policies; (3) specific sector policies (such as the building sector); and (4) more balanced national policies. A detailed comparison of the main characteristics of the three datasets is given in Supplementary Table 2. The main purpose of comparison is to find which classification information (the fields in bold in the table) of these three datasets can help us classify.

### Indicator selection and description

The GCCMPD aims to provide a multi-indicator dataset that can be researched and consulted by scholars in various fields, policy-makers, and various social organizations. We mainly chose the following indicators to meet different research needs: sector, instrument, objective, legally binding force, enforcement settings and jurisdiction. Due to the diverse sources of the GCCMPD, choosing a unified standard and definition is conducive to maintaining the consistency of the dataset.

- **Sector:** One of the characteristics of the GCCMPD is that it can be closely integrated with the carbon emission dataset, which requires sectoral classification of policies to match them with GHG emissions. Most mainstream GHG emissions datasets^46,47^ refer to the IPCC 2006 reporting guidelines^48^ for sectoral classification and generally attribute global GHG emission sources to five broad sectors^49^ of energy systems, industry, buildings, transport and AFOLU (agriculture, forestry and other land uses). For the above reasons, the GCCMPD also divides sectors into these five sectors, adopting the same sectoral classification standards as the carbon emissions dataset based on the EDGAR^50^, and the classification standards and descriptions are shown in Supplementary Table 3.
- **Instrument:** A taxonomy of policy instruments can help policy-makers formulate policy packages, provide possible options for policy practice, and better evaluate climate policies. Some taxonomies, such as the NATO scheme^51^ (A widely-used standard that categorizes policies based on the types of governing resources they rely on: Nodality, Authority, Treasure, and Organization.), represent general criteria and are less specific to a certain domain of climate mitigation. We chose the classification standard of the IPCC Fifth Assessment Report (AR5)^52^, which has the following advantages. First, the classification is authoritative. The classification criteria are summarized by climate experts based on a large amount of literature, reflecting the climate policies that the academic community focuses on. Second, after the modification from the Third Assessment Report (TAR)^53^ to the AR5, the classification has strong completeness and timeliness. Third, there are sector-level classifications of policy instruments for the above five sectors, which can help to understand and use them in conjunction with sectoral classifications. As a result, policy instruments are divided into seven categories: taxes, tradable allowances, subsidies, government provisions of public goods or services, information programmes, regulatory approaches, and voluntary actions. Detailed sector-level standards and examples can be found in Supplementary Table 4.
- **Objective:** The mitigation of climate change is only one of many public issues, and sometimes it is necessary to judge whether climate change has co-benefits and adverse side effects on society, the economy, and the environment. For almost the same reasons as “instruments”, we chose the AR5 taxonomy, which categorizes climate mitigation impacts on economic, social, and environmental objectives/issues. The detailed subobjective classification can be found in Supplementary Table 5.
- **Binding force:** Whether the same policy instrument is legally binding affects its effectiveness, which is also the core difference between hard law and soft law. The concept of soft law was originally common in the EU and international law^54^. At present, countries need to consider multiple goals while mitigating climate change. Therefore, soft law has become a flexible choice when making climate commitments cautiously. Both soft and hard laws have advantages and disadvantages. Hard law usually balances the interests of all parties and is more democratic but has a long promulgation cycle and high costs. Soft law is formulated and adopted more quickly, but at the same time, there are problems of poor consideration and they can be overly ambitious^55^. In addition, the soft law is a good complement to the hard law, and its functions can be divided into prelaw functions, postlaw functions, and para-law functions^55^. For soft law, we refer to the classification based on function (as an alternative to legislation) proposed by Senden, L^55^, which divides soft law into three main categories: preparatory and informative instruments, interpretative and decisional instruments, and steering instruments. For hard laws, we mainly consider the priority when two hard laws conflict, that is, the legal hierarchy^56^. We refer to the legal hierarchy summarized by Clegg, M. *et al*.^56^, combined with the legal hierarchy of the European Union, the United States and other countries, and divide hard law into three main categories: constitution, statutes/legislation, and regulations/rules. The classification rules and descriptions of soft law and hard law can be found in Supplementary Table 6, and some examples are shown in Supplementary Table 7.
- **Executive/legislative:** The enactment of policies by executive or legislative bodies can partially measure the durability of policies (assuming that executive measures can more easily be removed by new governments, while laws are more durable, given that their removal requires the approval of the legislative body), referring to Iacobuta *et al*.^9^ Policy durability has attracted attention^9^ because it affects the expectations of policy implementation objects. For example, expectations regarding the durability of policies may affect short-term and long-term decision-making judgements of enterprises, thereby impacting the effectiveness of policy implementation. What is slightly different is that, like the CCLW, the GCCMPD considers whether policies are enacted by the legislative or executive branch of the government on the basis of law or strategy. For example, China’s 14th five-year plan is classified as executive in the CCLW and GCCMPD but not legislative.
- **Jurisdiction**: In addition to classifying the scope of policy from an industrial perspective, such as sector, the classification of policy jurisdiction can help to better analyse the interaction between central and local governments^57^, international competition and multilateral cooperation^58^, and regional emissions governance^59^. Therefore, we divided policy jurisdictions into international, national, subnational area (the broader category with multiple provinces), and subnational (single state/provinces, municipality/city) jurisdictions.

### Data collection

Some challenges of data collection are that there are few websites that can be directly packaged and downloaded, the webpage rules of each data source are different, and the rules are constantly updated. First, important policy features were manually selected based on the characteristics of each website (all policies include at least title, content, and year). Then, Python toolkits such as Requests (an elegant and simple library allows you to send HTTP requests) and Selenium (is used to automate web browser interaction from Python) were used to obtain the data. All the raw data are stored in document form (CSV, EXCEL) and data platform form (MongoDB).

### Data processing

Through steps 1-12 of the data processing shown in Fig. 1, we constructed a reference (original) version of the training set data as of December 31, 2021. To ensure the consistency of manual annotation and reduce manual annotation errors, the classification information of the IEA, CP, and CCLW datasets was used as much as possible. As shown in Supplementary Table 2, among the main indicators of the GCCMPD, the preliminary classifications of sector, instrument, objective (including detailed classification of subsector, sector-instrument and subobjective), and jurisdiction can refer to the IEA, CP, and CCLW classification information. The two indicators of binding force and executive/legislative can only partially refer to the IEA, CP, and CCLW classification information. Therefore, additional legal keywords were constructed with reference to legal dictionaries, such as Black’s law dictionary (https://thelawdictionary.org/) and Duhaime’s law dictionary (http://www.duhaime.org/), to assist in classification. Therefore, these steps were intuitively divided into three categories: IEA, CP, and CCLW classification information (steps 2, 4, 6, and 11); IEA, CP, and CCLW information (steps 7, 8, and 9); and simple rules and mappings (remaining steps).

A detailed description of the processing steps can be found in the Supplementary Information (SI2); the mapping dictionary built based on IEA, CP, and CCLW can be found in Supplementary Table 8-16; and the keywords built with reference to the legal dictionaries can be found in Supplementary Table 17-18.

Although there are differences in the definitions of mitigation and adaptation, thus, the two are strongly related^60^. Many climate policies often involve both mitigation and adaptation policies, and different datasets differ in terms of whether the same climate policy involves mitigation or adaptation. The GCCMPD adopts a negative list method to construct keywords (Supplementary Table 19) that significantly characterizes adaptation policies and remove them (One of the keywords in Supplementary Table 19 appears in the policy title or content, then the policy is judged to be climate adaptation.) from the dataset.

Below are the descriptive statistics of the training set. The training set contains climate policy data from 199 entities (198 countries and the European Union). Table 2 illustrates the distribution of entities and policies on each continent. According to Table 2, the number of African, Asian, and European entities is the largest. For the number of policies, although the number of European entities is not the largest, European entities issued the most policies among the six continents. The average number of policies issued by each entity is the highest in Europe (89.96) and lowest in Africa (16.09).

**Table 2:** Distribution of Entities and Climate Policies.

Continent	Number of Entities	Number of Policies
North America	24	1644
South America	12	617
Europe	45	4048
Asia	48	2314
Africa	53	853
Oceania	17	610
Total	199	10086

There are two policies issued by the World Bank and the United Nations, which may not be assigned to a single continent. Please refer to https://www.iea.org/policies/11690-the-world-bank-carbon-capture-and-storage-capacity-building-trust-fund; and https://www.iea.org/policies/11688-climate-technology-centre-and-network-technical-assistance. Please refer to Supplement Table 20 for the entity list covered in training set of GCCMPD.

There are 10088 policies ranging from 1927 to 2021 (among them, several policies in the IEA dataset are scheduled to enter into force in 2025) included in our core dataset. As Table 3 shows, national climate policies make up approximately 90% of our sample, while international policies account for 3% and subnational policies for 7%. We divided the policies into five sectors and found that most of the policies targeted energy systems (3871) and that the fewest policies focused on AFOLU (838). There are also 3843 policies focused on the whole economic level rather than on a single sector or several other sectors. Policies are divided into seven types according to the instrument they apply. Most policies employ the government provision of public goods or services (7040) and regulatory approaches (4467), while only 121 policies use the tradable allowance instrument. For the binding force of the policies, soft laws constitute a greater share of the sample (69.73%), while hard laws constitute only 30.27% of the sample. Law/Act is the most popular hard law, while Preparatory Instruments is the most widely used soft law. The classification of co-benefit objectives is mainly economic objective, followed by social objective.

**Table 3:** Number of different types of climate policies.

Features	Number of Policies	Features	Number of Policies
**Jurisdiction**		**Binding force**	
International	343	**Hard Laws**	
National	9049	Constitution	11
Subnational	696	International Law	2
**Sectors**		Law/Act	1647
AFOLU	838	Decree/Order/Ordinance	571
Buildings	2466	Regulation/Directive/Decision	823
Industry	1509	**Soft Laws**	
Energy systems	3871	Preparatory Instruments	3862
Transport	2356	Informative Instruments	261
Whole Economics Level	3843	Interpretative Communications and Notices	15
**Instruments**		Decisional Notices and Communications	84
Government Provision of Public Goods or Services	7040	Decisional Guidelines, Codes and Frameworks	1179
Information Programmes	3519	Steering Instruments	1253
Regulatory Approaches	4467	Other Strategy Plan or Target	380
Subsidies	3590	**Objectives**	
Voluntary Actions	377	Economic	4476
Taxes	506	Social	2047
Tradable Allowances	121	Environmental	1350

Policies may belong to multiple sectors, multiple instruments, and multiple objectives. Thus, the sum of the policies in each sector (or with each instrument, each objective) may exceed the total sample size. Among the subnational policies, 694 are issued by single subnational entities (categorized as “SubNational”, while 2 are issued by multiple subnational entities (categorized as “Subnational area”).

As Table 4 shows, by combining these three datasets, the number of policies in each sector has almost doubled. All four datasets have a high share of energy sector policies. In addition, the integration of the construction sector and the AFOLU sector shows the benefits of coordination. The IEA dataset also has a high proportion of construction sector policies, while the CCLW dataset has a relatively high proportion of AFOLU sector policies. GCCMPD generates complementary advantages through the integration of these datasets, further highlighting the significant potential for complementarity^35^ between the IEA, CP and CCLW.

**Table 4:** Comparison of our dataset and the IEA, CP, and CCLW datasets in terms of sector coverage.

Sectors	GCCMPD	IEA	CP	CCLW
AFOLU	838(0.06)	46(0.01)	357(0.05)	437(0.11)
Buildings	2466(0.17)	1948(0.25)	956(0.14)	355(0.09)
Industry	1509(0.10)	671(0.09)	636(0.09)	359(0.09)
Energy systems	3871(0.26)	1893(0.24)	1812(0.27)	1105(0.28)
Transport	2356(0.16)	1217(0.15)	1048(0.16)	693(0.17)
Multisector	3843(0.26)	2114(0.27)	1909(0.28)	1029(0.26)

Since a policy can involve multiple sectors, the proportion here is calculated based on the sum of each column in the table.

### Manual check, verification, annotation

#### Check for duplicates

Although merging the three datasets can combine their respective strengths, there will be some duplication of policies, as the three datasets share many of the same sources. Duplicate policies do not affect analysis, such as policy coverage^9,11,12^ and policy sequencing^18,21^, but do affect analysis based on policy strength^23,24^. Based on the Best Match 25 algorithm (BM25^61^, a ranking algorithm based on probabilistic relevance framework for generating the similarity score between two texts), we used all policy titles as a corpus, grouped the data by country, year, and jurisdiction, and calculated the text similarity score between policies. Human checks combined with text similarity scores eventually revealed duplicate policies (Supplementary Fig. 3).

#### Verification and annotation

It is also necessary to manually check, verify and annotate each indicator. Based on the reference version, the manual labelling bias was reduced. During manual verification, we also found that there were some policy instruments and sectors that cannot be found directly from the policy content, and the reference version was used to assist the manual work in making more accurate annotations. After manual checking and correcting the dictionary mapping several times, we found that even graduate students majoring in energy and climate were prone to label mistakes.

Here are a few illustrations of how to manually check policy sectors. We judged whether the policies belonged to the energy system or building sector based on whether the home solar PV system was connected to the grid because self-sufficiency alone cannot be considered the energy supply. Heating and cooling generally refer to household heating and cooling appliances (buildings) rather than district heating (energy systems). Fossil fuel extraction (energy systems) depends mainly on how the carbon emissions dataset is classified. Standards for building parking spaces to promote the use of new energy vehicles are categorized as transport and building policies. Equipment safety and photovoltaics are considered industry policies. Biofuels are considered transport policies, biomass power generation is considered for energy systems, and the manufacture of biofuels may involve industry and AFOLU. It should be noted that climate policy data differ from carbon emissions data. It is very common for a policy to involve multiple sectors.

For the instrument indicator, the tax category is relatively easy to mislabel. To distinguish it from subsidies, new taxes and tax increases are classified as taxes, and tax reductions and accelerated depreciation are classified as subsidies. Since the tax classification of the IEA is based on whether a certain type of tax appears in the policy text, the tax reduction will also be recorded as a tax, so it needs to be manually corrected. In addition, grants, awards, R&D subsidies, etc., also need to be classified as subsidies or/and government provisions of public goods or services according to the content of the policy. Since the government provision of public goods or services involves capacity building and the removal of barriers, it includes the establishment of institutions, the provision of financial support, etc. Another thing to point out is that subsectors (sector-instrument and subobjective) are more detailed but not completely classified; that is, this does not mean that a policy classified as a certain sector (instrument or objective) will necessarily be further classified into a certain subsector (sector-instrument and subobjective) (Supplementary Figs. 4-6). For example, some energy plans mentioned several sectors but did not involve specific subsectors. Some government funds support renewable energy power generation without specifying whether it is investment, R&D support, or subsidies.

#### Search, verification, and supplementation of policy content

The policy content also requires many manual searches and verification. According to Table 5, many policy contents in the CP and IEA are missing or too short, and this will affect the effectiveness of manual checking indicators, machine learning model training, and topic models. We tried to find the content of the policy by searching the source webpage. For some countries whose official language is English (e.g., Canada), the source web page was invalid or incorrect, and for countries whose official language is not English (e.g., Argentina, Spain, Germany, Japan, etc.), there were language problems. We used Google Search (when the source web page was invalid or incorrect) in addition to optical character recognition (OCR) technology and Google Translate for scanned documents and language issues. Through the above efforts, the missing value of policy content has been greatly reduced, and the quality of policy content has improved (Table 5). In addition, manual verification also included translating the policy title and content into English and correcting the policy year and jurisdiction.

**Table 5:** Comparison of the original policy content and the policy content after manual search and supplementation for IEA, CP, and CCLW.

Datasets	Policy Content	Original policy content	After manual supplementation
IEA	Missing policy content	315	0
	Average length	118.00	139.40
	Number of short sentences (tokens < 10)	373	38
	Proportion of short sentences (tokens < 10)	5.82%	0.63%
CP	Missing policy content	1764	118
	Average length	38.14	136.85
	Number of short sentences (tokens < 10)	2383	150
	Proportion of short sentences (tokens < 10)	47.75%	6.85%
CCLW	Missing policy content	0	0
	Average length	125.01	149.27
	Number of short sentences (tokens < 10)	8	0
	Proportion of short sentences (tokens < 10)	0.42%	0%

### Dataset expansion

After the above data processing and manual inspection, a refined training dataset containing 10088 policies was formed (GCCMPD-IEA-CP-CCLW). However, similar to other datasets, the GCCMPD-IEA-CP-CCLW still had shortcomings, such as high labour costs, slow update speed, and difficulty expanding (some data sources do not have relevant indicator information and cannot form dictionary maps). In this section, we used several state-of-the-art natural language processing (NLP) and machine learning techniques to expand the dataset. As shown in Table 6, through the application of machine translation, multilabel classification, single-label classification, named entity recognition, text similarity, and topic models, dataset construction was completed only by relying on policy titles and content.

**Table 6:** Models and methods used for the GCCMPD.

Method/Package	Natural Language Processing (NLP) Task	GCCMPD indicator/purpose of model
Google translation	*Machine translation*	Uniform policy title and content in English
ClimateBERT	*Multilabel classification*	Sector, Instrument, Objective
	*single-label classification*	Binding force; Executive/legislative
“en_core_web_trf” & “countryinfo”	*Named Entity Recognition (NER)*	Jurisdiction
BM25 & “en_core_web_trf”	*Text similarity*	Automatically find duplicates
BERTopic	*Topic Modelling*	The topic of each policy

#### Multilabel and single-label classification

The GCCMPD’s indicators, sector, instrument, and objective are multilabel classifications, while binding force and executive/legislative are single-label classifications. We chose the ClimateBERT^62^ algorithm, which is based on the Bidirectional Encoder Representations from Transformers (BERT) model, because it has high performance^63,64^, relatively low fine-tuning cost compared with large language models such as Generative pretrained transformer (GPT) or Large Language Model Meta AI (Llama), and concentration on climate change (pretrained on the text corpus of abstracts of climate-related research papers, corporate and general news, and company reports, and because it is currently the latest model in the field of climate change), and it is widely used in existing studies^65,66^. By comparing ClimateBERT and the traditional machine learning models logistic regression classifier (LR), naïve Bayes (NB), and support vector machine (SVM) with ClimateBERT, the results (Supplementary Table 21–25) demonstrate the advantages of ClimateBERT in the climate field. The detailed model training details and comparisons can be found in the Supplementary Information (SI3).

#### Named entity recognition (NER)

Judging climate policy jurisdictions by identifying states/provinces and countries that appear in policies through NER is an intuitive and highly interpretable method. Specifically, we first used the Roberta-based^67^ “en_core_web_trf” model to identify geopolitical entities and then use “countryinfo” to distinguish “state/province” or “country”. Furthermore, the “Subnational area” and “International” were determined according to the number of different cities and countries in a policy (Supplementary Table 26).

#### Text similarity

**Fig. 2:**
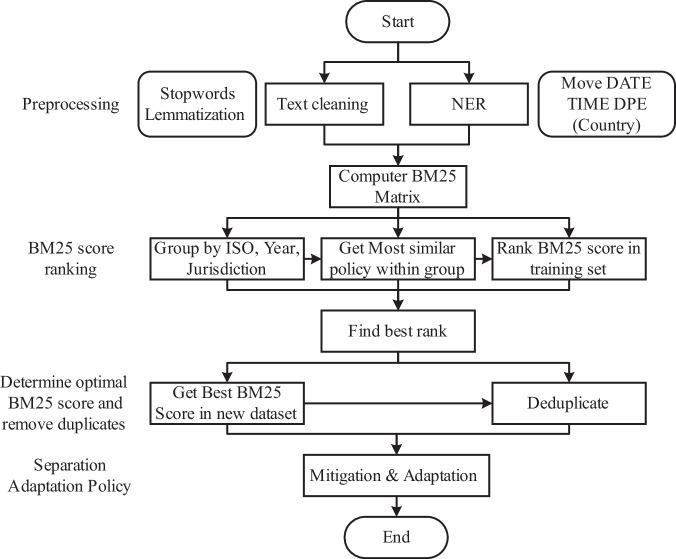
Machine learning processes for identifying duplicate policies.

In the GCCMPD-IEA-CP-CCLW, after calculating the BM25 score, we determined the duplication of policies from different sources by manual checking in groups. However, for data expansion, two issues needed to be considered: 1. When the amount of data increases rapidly, methods based on a large amount of labour are no longer applicable. 2. How can we objectively judge whether two policies are the same? Some text similarity methods, such as bag-of-words/TFIDF combined with cosine similarity, can obtain a standardized value, but thresholds such as 0.8 still need to be set subjectively, and the methods are relatively rough. We still used the BM25 algorithm and combined the ideas of model training and evaluation to automatically find duplicates (Fig. 2). Finally, based on the optimal ranking of 6061 and an F1 score of 0.8 (Supplementary Fig. 7), the optimal BM25 score was determined. A policy with a similarity score greater than the optimal BM25 score was judged to be a duplicate policy. The technical details and detailed instructions can be found in the Supplementary Information (SI2).

#### Topic model

Policy topic analysis can aid in understanding policy evolution^17^ and has become a new branch of research in the field of public policy. Currently, BERTopic^68^ is a state-of-the-art topic model that effectively uses word embedding to transform input documents into numerical representations, dimensionality reduction to reduce the dimensionality of the embeddings to a workable dimensional space and clustering similar embeddings to obtain topic representations through c-TF-IDF (an adjusted TF-IDF to work on cluster/categorical/topic level instead of document level). The GCCMPD provides 4 topic models for different analysis needs, namely, GCCMPD-IEA-CP-CCLW-Topic, GCCMPD-EXPAND-Topic (counting multilateral policies as multiple), GCCMPD-Topic (counting multilateral policies as one) and GCCMPD-EXCEPT-ECOLEX-Topic (excluding ECOLEX). The specific model details can be found in the Supplementary Information (SI2).

## Data Records

**Fig. 3:**
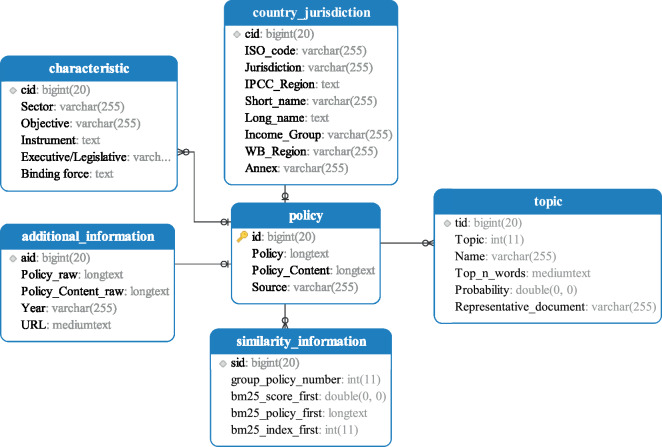
Structure of the GCCMPD core data.

To facilitate the use of various scientific researchers, government staff, and personnel of international organizations, the GCCMPD provides three forms of data storage: MySQL, MongoDB, and EXCEL. All data records have been uploaded through Figshare: 10.6084/m9.figshare.22590028.v2^69^. The relational data management system (MySQL) can well reflect the composition of core data (Fig. 3):

- **Policy:** contains all deduplicated climate mitigation policies (policy ID, policy title and content translated into English, policy source).
- **Additional information:** includes the year the policy was published, the original title and content of the policy, and the policy source document web page.
- **Characteristic:** contains indicators, sector, objective, instrument, executive/legislative, and binding force, for each policy.
- **Similar information:** provides the total number of policies in each policy group, the index, the BM25 score, and the title of the most similar policy in the group.
- **Country and Jurisdiction:** provides information about the country where the policy was adopted. The table is also linked to external countries’ economic (e.g., GDP, imports and exports), social (e.g., population, democracy), political (e.g., left-wing or right-wing, federal), and energy (e.g., primary energy usage) and characteristic dataset media.
- **Topic**. provides the topic category of each policy, topic name, the most representative words under this topic, the probability that the policy belongs to this topic, and whether it is a representative policy of this topic.

In addition to the core data, the GCCMPD also retains the processing results of each step. Supplementary Table 27 lists some important result files and their descriptions. These files are all stored in EXCEL.

## Technical Validation

The data quality of the GCCMPD is affected by several factors. The first is the data source. The comprehensiveness of information from different data sources varies greatly, which directly affects the model training ability. The second is the reliability of the annotation and training sets and the training ability of the model. Since the construction idea of the GCCMPD is to first form a training set through manual labelling and then extrapolate to a richer data source, the quality of the annotation and training set is highly important. We took the following steps to enhance the data quality:

- **Manual data retrieval:** The determination of data sources was performed through high-quality literature, Google searches, and data sources from authoritative datasets. The requirements for the data sources were as follows: 1. Include the title and content of the policy, and there should not be too many missing values; 2. Include the year of the policy; 3. The entity information of the policy release is contained as much as possible.
- **Manual annotations and checks:** We compared the annotations of PhD students in multiple climate and energy fields (with each person annotating a small number of fields) and found that the differences in their annotations were concentrated in several places (Manual check, verification, annotation section). Although we had given classification criteria and explained where it is easy to misclassify, manual annotation was inevitably subjective. Fortunately, objectivity and consistency can be guaranteed through dictionary mapping. It can be considered that IEA, CP, and CCLW data are the results of authoritative experts. We regard them as real values and the GCCMPD data as predicted values and compare the results (It is called comparison because the prediction result is not based on the model, but only draws on the metrics to give a more intuitive comparison result) of the two (Tables 7–11, for the comparison of subsectors, sector instruments, and subobjectives. See Supplementary Table 28-30 for additional information). The resulting recall rates were generally high, even close to 1, which means that we fully refer to the results of dictionary mapping. Some categories with lower recall results (such as Other Strategy Plan or Target) indicate that we later focused on checking such results. Some results with low precision rates were mostly because the original policy data source did not have corresponding keyword labels, and we annotated them during manual verification and inspection. The precision rate of the taxes category was not high because we checked and found that the classification standard of the IEA dataset was different from that of the GCCMPD dataset, while the precision rate of the subsidies category was not high because keywords such as “Award” and “Grants” cannot determine whether they are subsidies. Therefore, we did not construct a dictionary for relevant keywords and instead we added annotations through manual inspection. For the manual inspection of duplicate data, we also tried to be as detailed as possible and record the specific corresponding relationship of policy duplication.

**Table 7:** Comparison between dictionary mapping and manual verification of policy instrument results.

Instruments	Dictionary Mapping vs. Manual check		
	Precision	Recall	F1
Tradable Allowances	0.63	1.00	0.77
Regulatory Approaches	0.72	1.00	0.84
Taxes	0.18	0.96	0.30
Information Programmes	0.61	0.97	0.75
Government Provision of Public Goods or Services	0.83	1.00	0.90
Voluntary Actions	0.98	1.00	0.99
Subsidies	0.37	0.99	0.54
Macro Average	0.62	0.99	0.73

**Table 8:** Comparison between dictionary mapping and manual verification of policy sector results.

Sector	Dictionary Mapping vs. Manual check		
	Precision	Recall	F1
Multisector	0.94	0.91	0.92
Energy systems	0.86	0.96	0.91
Buildings	0.92	0.92	0.92
Industry	0.79	0.92	0.85
Transport	0.95	0.96	0.96
AFOLU	0.87	0.99	0.92
Macro Average	0.89	0.94	0.91

**Table 9:** Comparison between dictionary mapping and manual verification of policy objective results.

Objective	Dictionary Mapping vs. Manual check		
	Precision	Recall	F1
Environmental	0.75	1.00	0.86
Social	0.83	0.99	0.90
Economic	0.96	1.00	0.98
Macro Average	0.85	0.99	0.91

**Table 10:** Comparison between dictionary mapping and manual verification of binding force results.

Binding force	Dictionary Mapping vs. Manual check		
	Precision	Recall	F1
Constitution	1.00	1.00	1.00
International Law	1.00	1.00	1.00
Law/Act	0.94	1.00	0.97
Decree/Order/Ordinance	0.94	1.00	0.97
Regulation/Directive/Decision	0.95	0.99	0.97
Preparatory Instruments	0.96	0.99	0.97
Informative Instruments	0.80	0.94	0.86
Interpretative Communications and Notices	0.87	0.72	0.79
Decisional Guidelines, Codes and Frameworks	0.97	0.96	0.97
Decisional Notices and Communications	0.75	0.74	0.75
Steering Instruments	0.97	0.91	0.94
Other Strategy Plan or Target	1.00	0.62	0.76
Macro Average	0.93	0.91	0.91

**Table 11:** Comparison between dictionary mapping and manual verification of executive/legislative results.

Executive/legislative	Dictionary Mapping vs. Manual check		
	Precision	Recall	F1
Executive	0.98	1.00	0.99
Legislative	1.00	0.90	0.94
Macro Average	0.99	0.95	0.97
- **Enhanced model training capabilities:** Based on the use of state-of-the-art models, we improved model learning by translating non-English policies into English and checking and completing policy content. In terms of manually checking policy content, we selected content that could be used to summarize the policy, such as goals, purposes, and objectives, as well as information that could be used to assist in judging the legal category.

After the GCCMPD data are made public, we will also enhance the data sources and correct errors through interactions with data users.

Nonetheless, our dataset still has flaws, one of which is that our dataset does not contain adaptation policies. Climate adaptation is a very important field^70^, especially for countries with high climate vulnerability and high mitigation costs. Future research can improve our study by constructing an adaptation policy dataset.

### Supplementary information

Supplementary figures

## Data Availability

Code, dataset and some intermediate results are freely available on the following GitHub repository: https://github.com/HUANGZHIHAO1994/GCCMPD-climate-policy-dataset.
