# Supplementary material for: Harmonizing existing climate change mitigation policy datasets with a hybrid machine learning approach
Source: Sci Data. 2024 Jun 4;11:580. doi: 10.1038/s41597-024-03411-z (PMC11150542; doi:10.1038/s41597-024-03411-z)
Supplement: Supplementary file 1 — Supplementary figures [file 41597_2024_3411_MOESM1_ESM.docx]

Supplementary Information for

**Harmonizing Existing Climate Change Mitigation Policy Datasets with a Hybrid Machine Learning Approach**

Libo Wu^1,2,3,4,*^ , Zhihao Huang^1^ , Xing Zhang^1^, Yushi Wang^1^

1. School of Data Science, Fudan University, Shanghai, 200433, China

2. Institute for Big Data, Fudan University, Shanghai, 200433, China

3. School of Economics, Fudan University, Shanghai, 200433, China

4. Shanghai Institute for Energy and Carbon Neutrality Strategy, Fudan University, Shanghai, 200433, China

*Corresponding author(s): Libo Wu (wulibo@fudan.edu.cn)

**Supplementary Information**

[Supplementary Information SI 1: Search terms to search in the Scopus 4](#_Toc163736153)

[Supplementary Information SI 2: Detailed description of the data processing steps 4](#_Toc163736154)

[Supplementary Information SI 3: Detailed technical introduction and model comparison 6](#_Toc163736155)

**List of Figures**

[Fig. 1 Number of articles related to climate policy. 8](#_Toc165751474)

[Fig. 2 Topic distribution of climate policy literature. 9](#_Toc165751475)

[Fig. 3 Number and duplication of climate policy datasets. 10](#_Toc165751476)

[Fig. 4 Policy instrument and sector instrument. 11](#_Toc165751477)

[Fig. 5 Policy sector and subsector. 12](#_Toc165751478)

[Fig. 6 Policy objective and subobjective. 13](#_Toc165751479)

[Fig. 7 Identification of the optimal BM25 score and the optimal rank. 14](#_Toc165751480)

**List of Tables**

[Table 1. The selection criteria for climate-related policy data. 15](#_Toc165768272)

[Table 2. Summary and comparison of IEA, CP, and CCLW. 16](#_Toc165768273)

[Table 3. GCCMPD Sector Classification Reference. 18](#_Toc165768274)

[Table 4. GCCMPD Instrument Classification Reference. 24](#_Toc165768275)

[Table 5. GCCMPD Objective Classification Reference. 29](#_Toc165768276)

[Table 6. GCCMPD Binding Force classification reference. 30](#_Toc165768277)

[Table 7. GCCMPD Binding force Classification examples. 32](#_Toc165768278)

[Table 8. Dictionary mapping of IEA Instruments and Sector-Instruments. 33](#_Toc165768279)

[Table 9. Dictionary mapping of Climate Policy Instruments and Sector-Instruments. 40](#_Toc165768280)

[Table 10. Dictionary mapping of CCLW Instruments and Sector-Instruments. 43](#_Toc165768281)

[Table 11. Dictionary mapping of IEA Sector and Subsector. 45](#_Toc165768282)

[Table 12. Dictionary mapping of Climate Policy Sector and Subsector. 48](#_Toc165768283)

[Table 13. Dictionary mapping of CCLW Sector and Subsector. 49](#_Toc165768284)

[Table 14. Dictionary mapping of IEA Objective and Subobjective. 50](#_Toc165768285)

[Table 15. Dictionary mapping of Climate Policy Objective and Subobjective. 51](#_Toc165768286)

[Table 16. Dictionary mapping of CCLW Objective and Subobjective. 52](#_Toc165768287)

[Table 17. Dictionary mapping of policy titles on Hard law and Soft law. 57](#_Toc165768288)

[Table 18. Dictionary mapping of policy contents on Hard law and Soft law. 59](#_Toc165768289)

[Table 19. Adaptation keywords. 60](#_Toc165768290)

[Table 20. Entities covered in the training set of GCCMPD. 61](#_Toc165768291)

[Table 21. Performance of Instruments Multi-label Classification Model. 62](#_Toc165768292)

[Table 22. Performance of Sector Multi-label Classification Model. 63](#_Toc165768293)

[Table 23. Performance of Objective Multi-label Classification Model. 64](#_Toc165768294)

[Table 24. Performance of Binding force Single-label Classification Model. 65](#_Toc165768295)

[Table 25. Performance of Executive/legislative Single-label Classification Model. 66](#_Toc165768296)

[Table 26. Criteria for judging jurisdictions based on named entity recognition results. 67](#_Toc165768297)

[Table 27. Key Results Documents from GCCMPD. 68](#_Toc165768298)

[Table 28. Comparison of dictionary mapping and manual checking results on Sector-Instrument. 69](#_Toc165768299)

[Table 29. Comparison of dictionary mapping and manual checking results on Subsector. 71](#_Toc165768300)

[Table 30. Comparison of dictionary mapping and manual checking results on Subobjective. 72](#_Toc165768301)

Supplementary Information SI 1: Search terms to search in the Scopus

We use the following search terms to search in the Scopus: TS = (("climate polic*" OR "climate strateg*" OR "climate legislation*" OR ("policy database" AND "climate change")) OR ("polic*" AND ("greenhouse gas" OR "GHG" OR "CO2" OR "carbon emissions")) OR (("energy polic*" AND "climate change") OR ("policy design" AND "climate change")) OR (("companion policies" AND "climate")) OR (("surve*" AND "climate action")))

Supplementary Information SI 2: Detailed description of the data processing steps

Step 1: Use the "Country", "Year", "Jurisdiction", "Policy Title" information to simply determine the duplicate.

Step 2: According to SI Tables 11-13, the relevant classifications of IEA, CP, and CCLW are mapped to the sector and subsector^[[1]](#footnote-1)^ standards of SI Table 3 adopted by GCCMPD.

Step 3: According to the country ISO information, regional information is marked with the regional standards of IPCC and World Bank (WB), and income information is marked according to the income group of WB. Among them, the IEA data set only provides country names, so many rule-based judgments need to be added.

Step 4: According to SI Tables 8-10 and sector information in step 2, the relevant classifications of IEA, CP, and CCLW are mapped to the instrument and sector-instrument standards of SI Table 4 adopted by GCCMPD.

Step 5: According to country ISO information in step 3, further classify Annex I and Non-Annex I.

Step 6: According to SI Tables 14-16, the relevant classifications of IEA, CP, and CCLW are mapped to the objective and subobjective standards of SI Table 5 adopted by GCCMPD.

Step 7-8: For binding force, according to some keywords in policy title (SI Table 17), such as Constitution, Law, Decree, Resolution, etc., the classification task has been completed. The soft law, especially the soft law with post-law function, needs to be classified in combination with the hard law that appears in the policy content (SI Table 18). Based on the keywords in SI Table 17 and 18, a rule-based method is used to classify binding force of policies that cannot refer to IEA, CP, and CCLW. In addition to similar processing as above, Executive/legislative in step 8 refer to the hard-law and soft-law classification information completed in step 7.

Step 9: GCCMPD focuses on climate mitigation policies and therefore hopes to distinguish only those policies that are clearly climate adaptation, i.e., a negative list (SI Table 19) approach is used to identify and eliminate policies with obvious climate adaptation characteristics. Specifically, if one of the keywords in SI Table 19 appears in the policy title or content, then the policy is judged to be climate adaptation.

Step 10: The steps before step 10 process IEA, CP, and CCLW separately. Step 10 merges the processed results.

Step 11: Jurisdiction classification is completed through mapping and judgment methods. For example, IEA's “Regional” is mapped to Subnational area, and CP's “Subnational region” is mapped to Subnational.

Step 12: Based on BM25 algorithm^[[2]](#footnote-2)^, we use all policy titles as a corpus, group the data by country, year, and jurisdiction, and calculate the text similarity score between policies.

These process steps, such as unifying country codes, distinguishing between Annex I and non- Annex I countries, and realizing different regional classifications, are all for matching data from other sources (economic, political), so as to better link downstream policy analysis tasks.

Supplementary Information SI 3: Detailed technical introduction and model comparison

**Classification Model:**

Bidirectional Encoder Representations from Transformers (BERT) is a large pre-trained language model based on Transformer bidirectional training, and can be fine-tuned on text classification and other NLP tasks. It is still state-of-the-art in many NLP tasks^64^. ClimateBERT^65^, based on DistilRoBERTa^66^, additionally pretrained on the text corpus of abstracts of climate-related research papers, corporate and general news, and company reports is currently the latest model in the field of climate change. For the classification task, we combine information from both policy title and policy content, using 85% of the dataset for training and 15% for testing. For traditional machine learning models Logistic Regression classifier (LR), Naïve Bayes (NB), Support Vector Machine (SVM), before training, we use TFIDF^[[3]](#footnote-3)^ to extract features and PCA^[[4]](#footnote-4)^ method to reduce dimensionality. For BERT and ClimateBERT, we choose the bert-base-uncased and distilroberta-base-climate-f models for training. For binding force, we use the rule-based method to judge the constitution and international law, because the training samples are too few (less than 5), and the rules are enough to distinguish these two categories. Based on the training results, we find that ClimateBERT does outperform in climate-related policy domains (Supplementary Table 21-26). In these classification tasks, the number of labels for more than half of the tasks is greater than 5, and we use a relatively strict metric (macro F1 score) for small sample categories^[[5]](#footnote-5)^. Therefore, the F1 score of ClimateBERT in all classification tasks exceeds 0.7, and the F1 score is low only in the category with too small sample size. Such results are acceptable and can be used as the baseline for this classification problem. In the classification of some climate-specific fields (such as tradable allowance), ClimateBERT has shown an obvious advantage, while in other fields (such as binding force), Bert is more dominant, but the gap between the two model is small. The reason for the above results is that the corpus trained by Bert is more diverse and the parameters are more than 1.3 times that of ClimateBERT.

**Text similarity:**

- **Preprocessing.** Before calculating the BM25 score, we first clean the policy title, remove stop words (including punctuation, symbols, etc.) and lemmatization, and identify and remove time and country^[[6]](#footnote-6)^ information through NER.
- **BM25 score ranking.** We group policies by year, country, and jurisdiction, and find the most similar policy for each policy in the group by sorting the BM25 score, and record the BM25 score as the similarity score of this policy. We regard judging duplication as a prediction task, sort by the above similarity score in descending order, and given a certain rank, all policies before this rank are marked as duplicates.
- **Find optimal rank.** GCCMPD-IEA-CP-CCLW contains raw data, deduplicated data and duplicated data. Due to careful manual checking, it is recorded in detail which policy/policies each duplicate policy is duplicated with, so we regard it as ground truth. The best rank is determined by the maximum F1 score (Fig. 3). Finally, the optimal rank (6061) is determined by the maximum F1 score (0.8).
- **Determine optimal BM25 score in new data and remove duplicates.** After determining the optimal rank, for the expanded data, repeat the above preprocessing and BM25 score sorting steps^[[7]](#footnote-7)^. Since GCCMPD-IEA-CP-CCLW is a subset of GCCMPD, the optimal BM25 score in the new data set can be determined according to the optimal rank in the subset. A policy with a similarity score greater than the optimal BM25 score is judged to be a duplicate policy.
- **Separation Adaptation Policy.** Mitigation and adaptation policies are treated in the same way as GCCMPD-IEA-CP-CCLW.

**Topic Model:**

BERTopic is very modular. In addition to choosing embeddings, dimensionality reduction and clustering models according to specific problems, it can also calculate the topic representations at each time step to realize the dynamic topic modeling, and realize hierarchy topic modeling by calculating the c-TF-IDF matrix similarity. GCCMPD obtains topics by using ClimateBERT embedding, UMAP dimensionality reduction, and HDBSCAN clustering. Since C-TF-IDF is a method based on statistical word frequency, we additionally eliminated entities (such as PERCENT, PERSON, etc.), and removed many physical units, abbreviations, etc.

GCCPMD assigns policies to each country for policies involving multiple countries (such as multi-country cooperation in research and development of green technologies, etc.). The reason is that jurisdictions can distinguish it from national policy, and applications such as policy query, policy quantity-based analysis, policy coverage analysis, etc. will not be affected. However, policy topics will obviously be affected by the above-mentioned treatment. There are different ways of understanding this. One way of interpretation is that multilateral cooperation policies show great influence, so it is reasonable to split a policy into multiple identical ones. There is another way of interpreting that multilateral cooperation can only be counted once in topic statistics. In this regard, GCCMPD provides 4 topic models for different analysis needs, namely GCCMPD-IEA-CP-CCLW-Topic, GCCMPD-EXPAND-Topic (counting multilateral policies as multiple), GCCMPD-Topic (counting multilateral policies as one) and GCCMPD-EXCEPT-ECOLEX-Topic (ECOLEX is excluded).


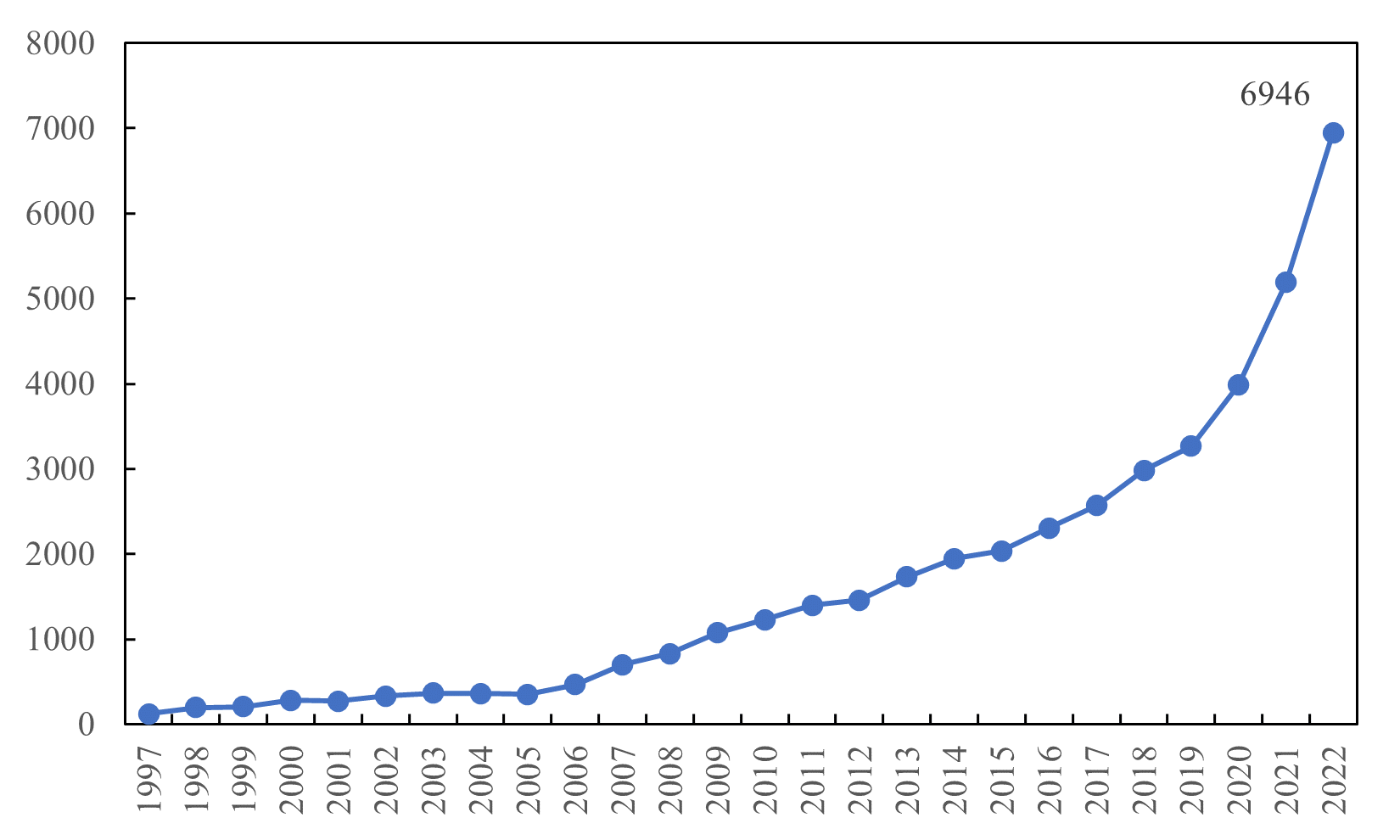


Fig. 1 Number of articles related to climate policy.

| 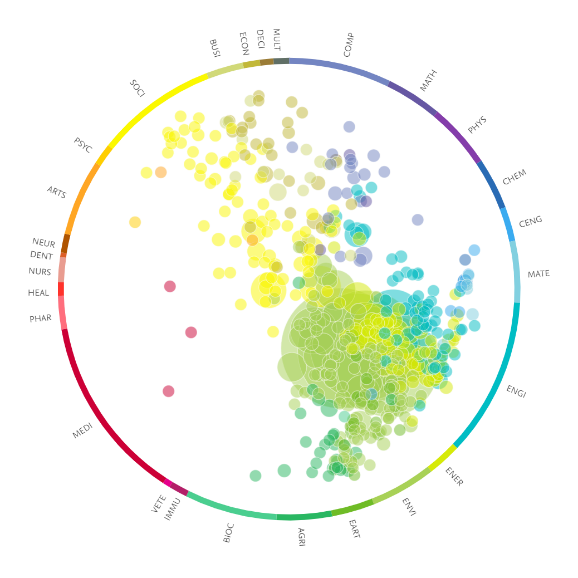 | 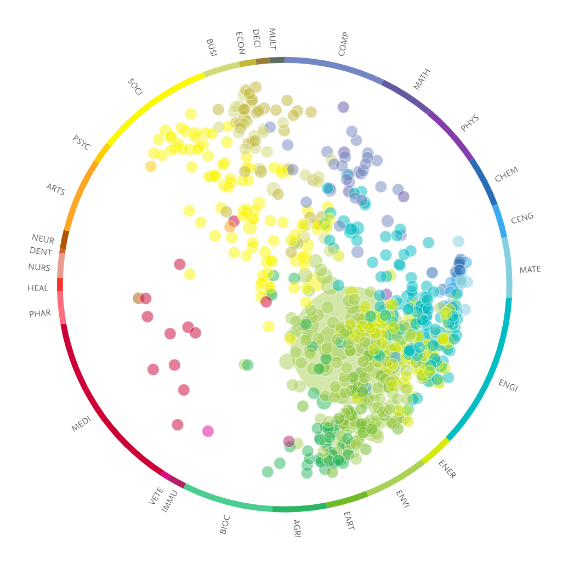 |
| --- | --- |
| 2018 Climate Policy Literature Topics | 2022 Climate Policy Literature Topics |

Fig. 2 Topic distribution of climate policy literature.


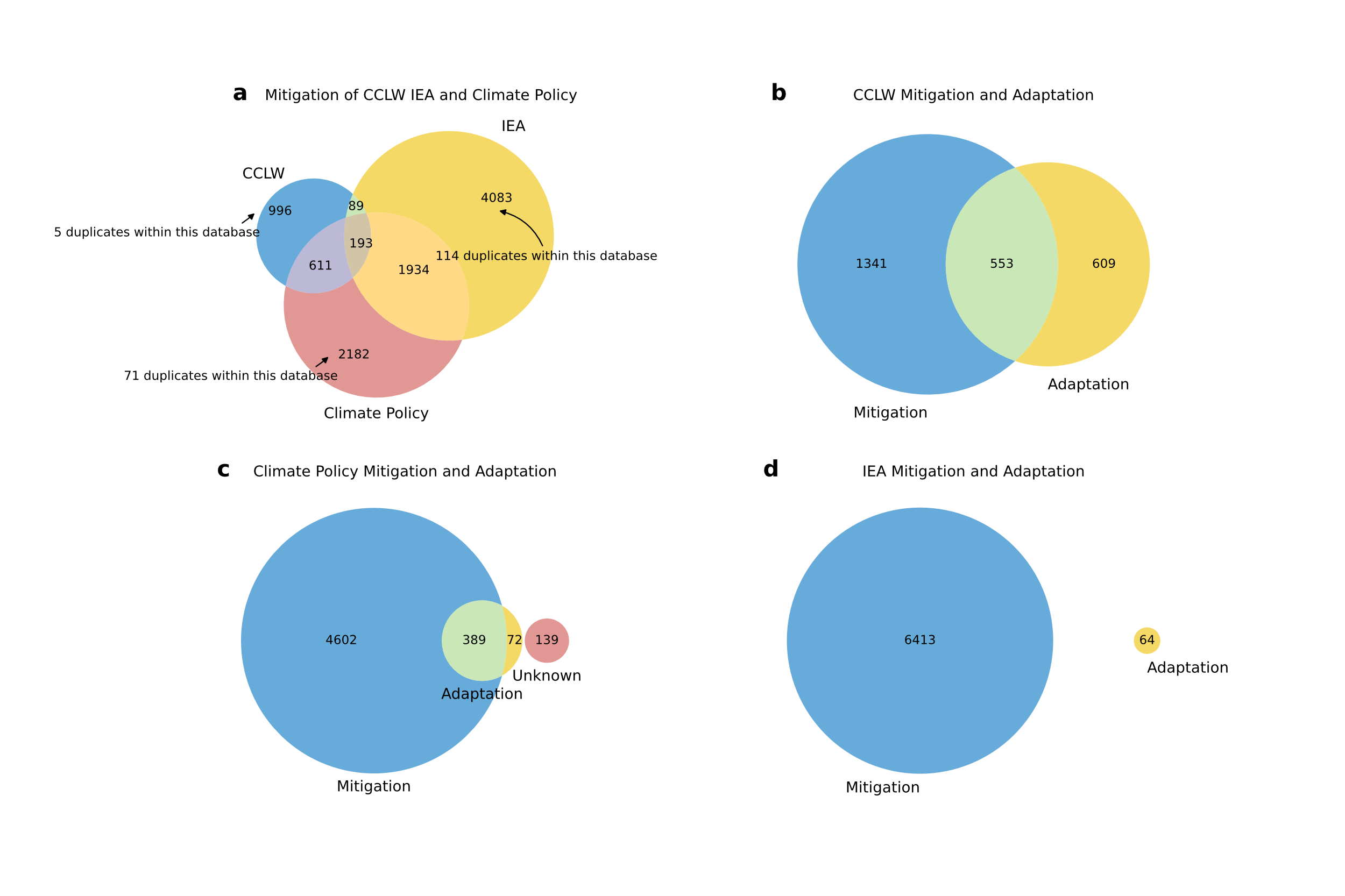


Fig. 3 Number and duplication of climate policy datasets.

a, Climate mitigation policies of CP, CCLW, and IEA datasets. Through deduplication, 10088 (996+4083+2182+611+89+1934+193). b-d, Number of climate mitigation and adaptation policies of CCLW (b), CP (c), IEA (d). As of December 31, 2021, CCLW has a total of 2,503 policies, CP has a total of 5,207 policies (including 5 of completely repeated data), and IEA has a total of 6,536 policies (including 59 of completely repeated data).


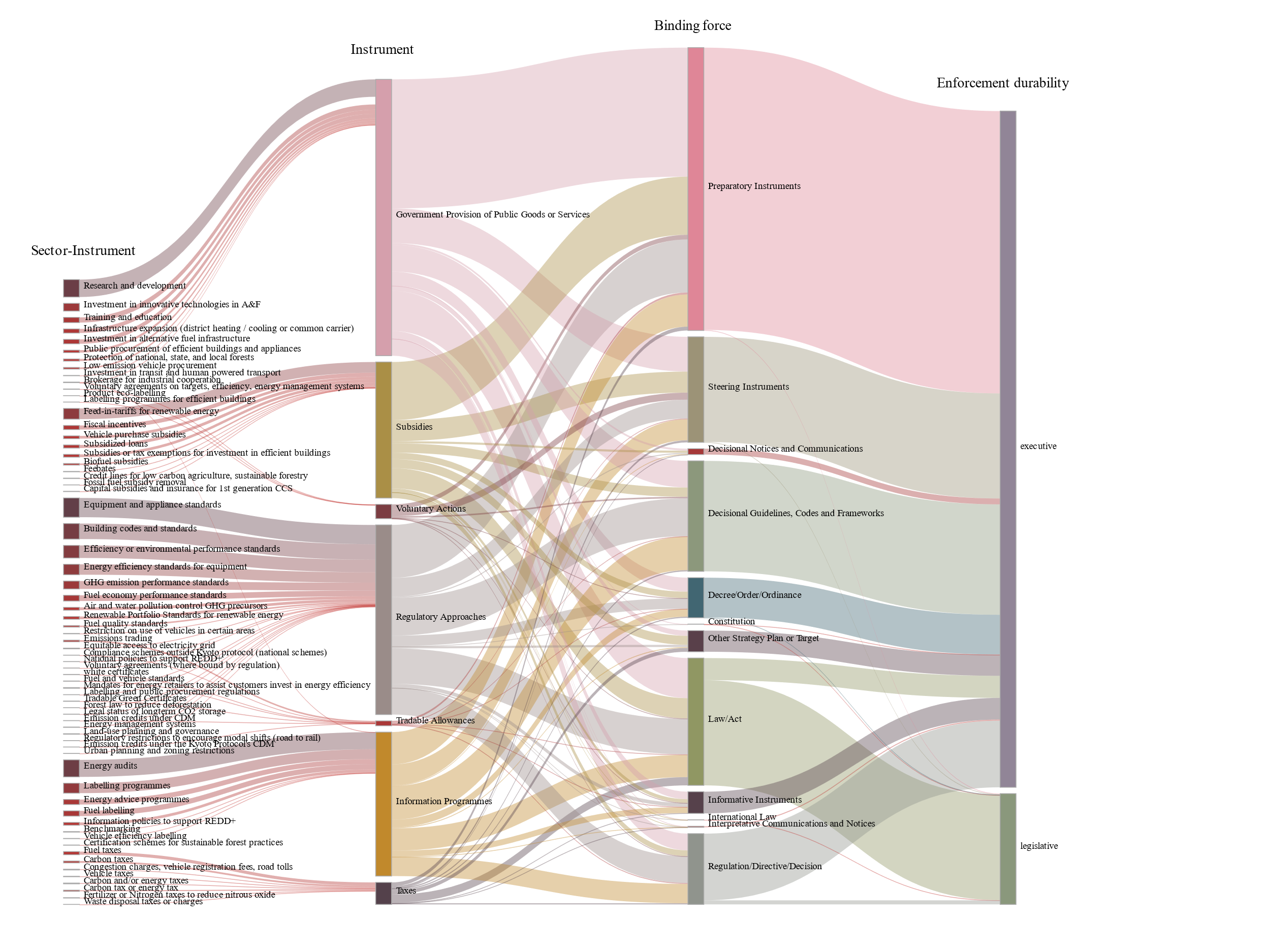


Fig. 4 Policy instrument and sector instrument.

The length of the bar represents the quantity, and the flow direction represents the proportion. A blank flow direction indicates that a certain item cannot be accurately classified through dictionary mapping and policy text information. From left to right, each bar graph of the Sankey diagram represents Sector-Instrument, Instrument, Binding force, and Executive/legislative.


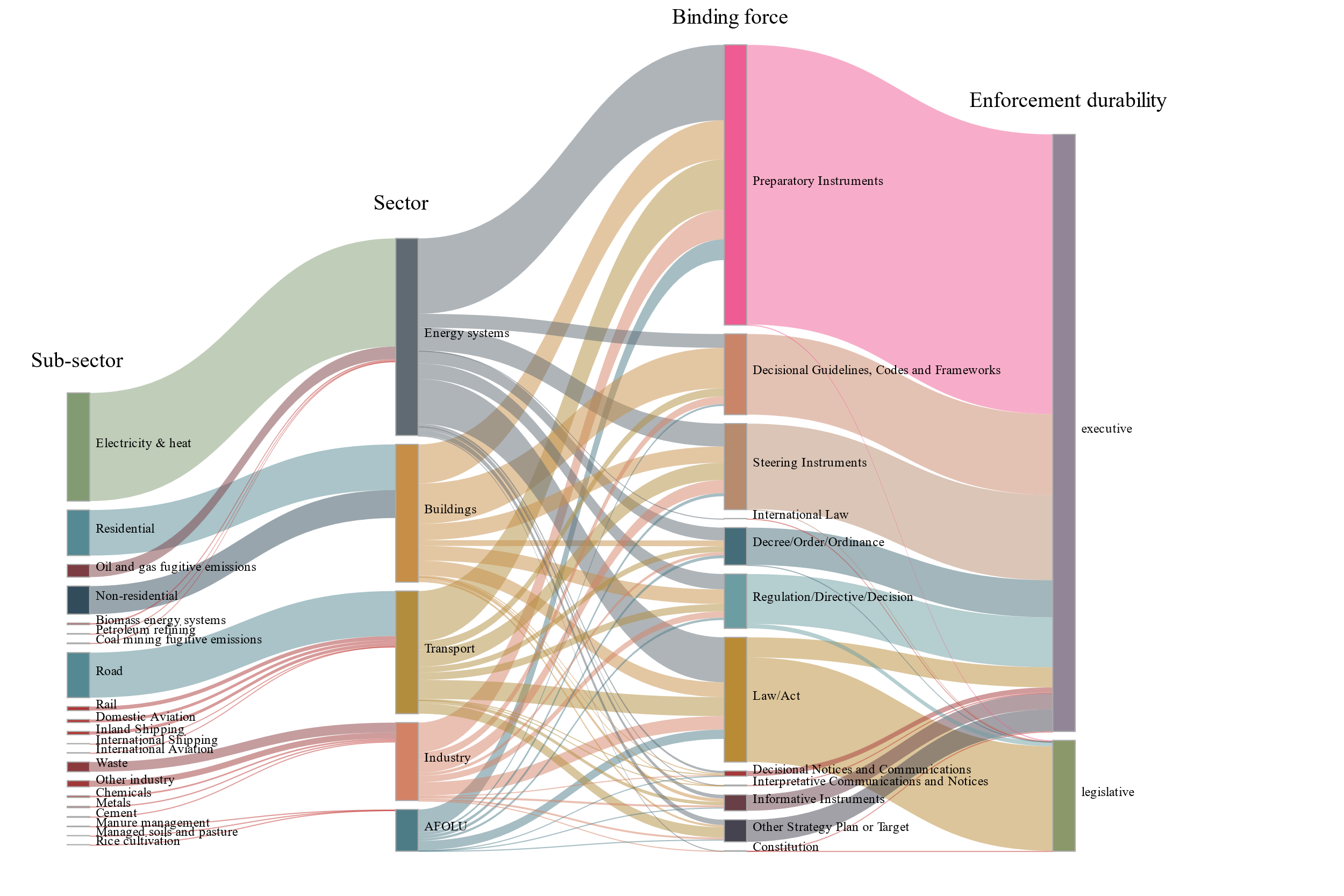


Fig. 5 Policy sector and subsector.

The length of the bar represents the quantity, and the flow direction represents the proportion. A blank flow direction indicates that a certain item cannot be accurately classified through dictionary mapping and policy text information. From left to right, each bar graph of the Sankey diagram represents Sub-sector, Sector, Binding force, and Executive/legislative.


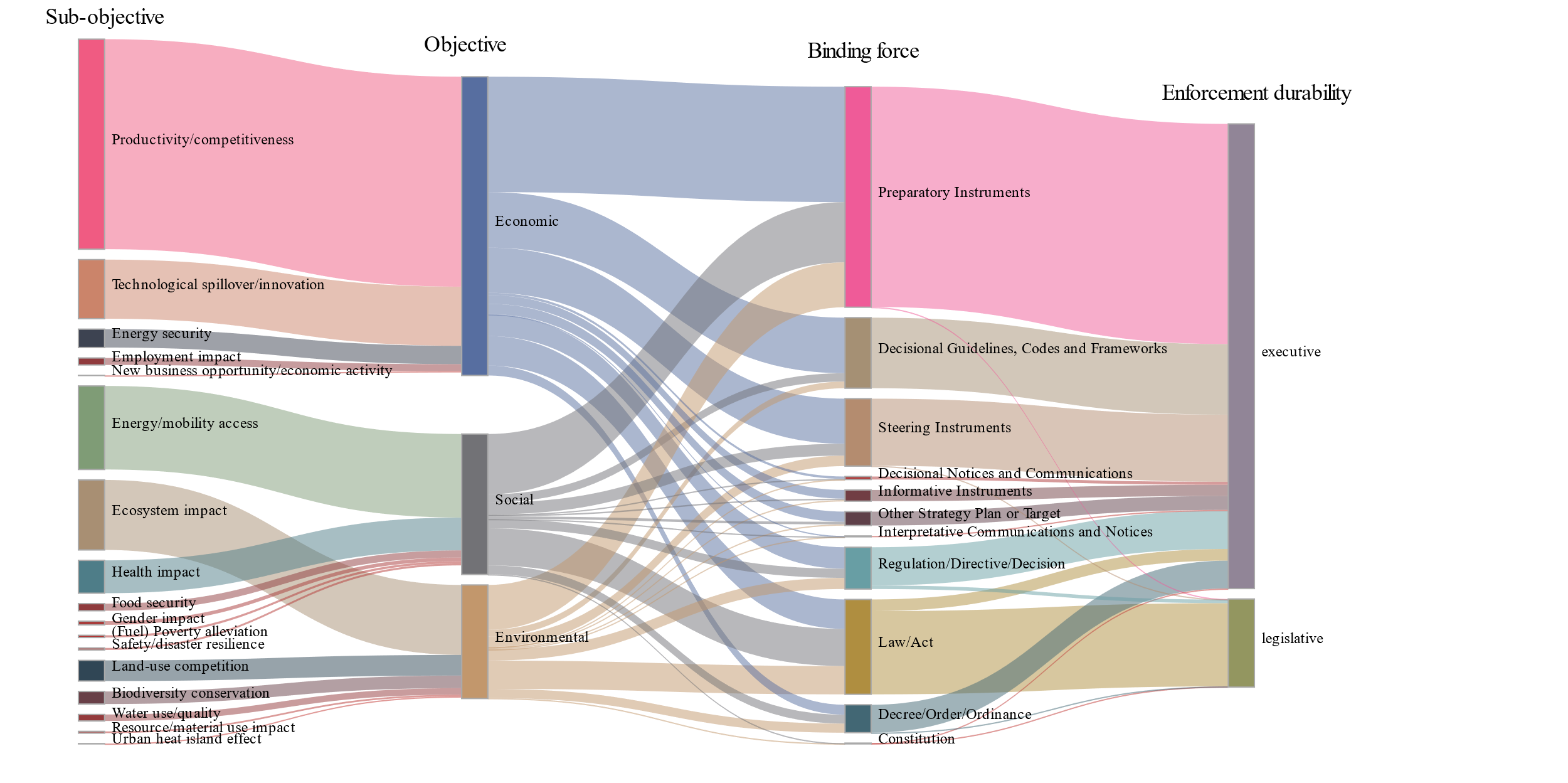


Fig. 6 Policy objective and subobjective.

The length of the bar represents the quantity, and the flow direction represents the proportion. A blank flow direction indicates that a certain item cannot be accurately classified through dictionary mapping and policy text information. From left to right, each bar graph of the Sankey diagram represents Sub-objective, Objective, Binding force, and Executive/legislative.


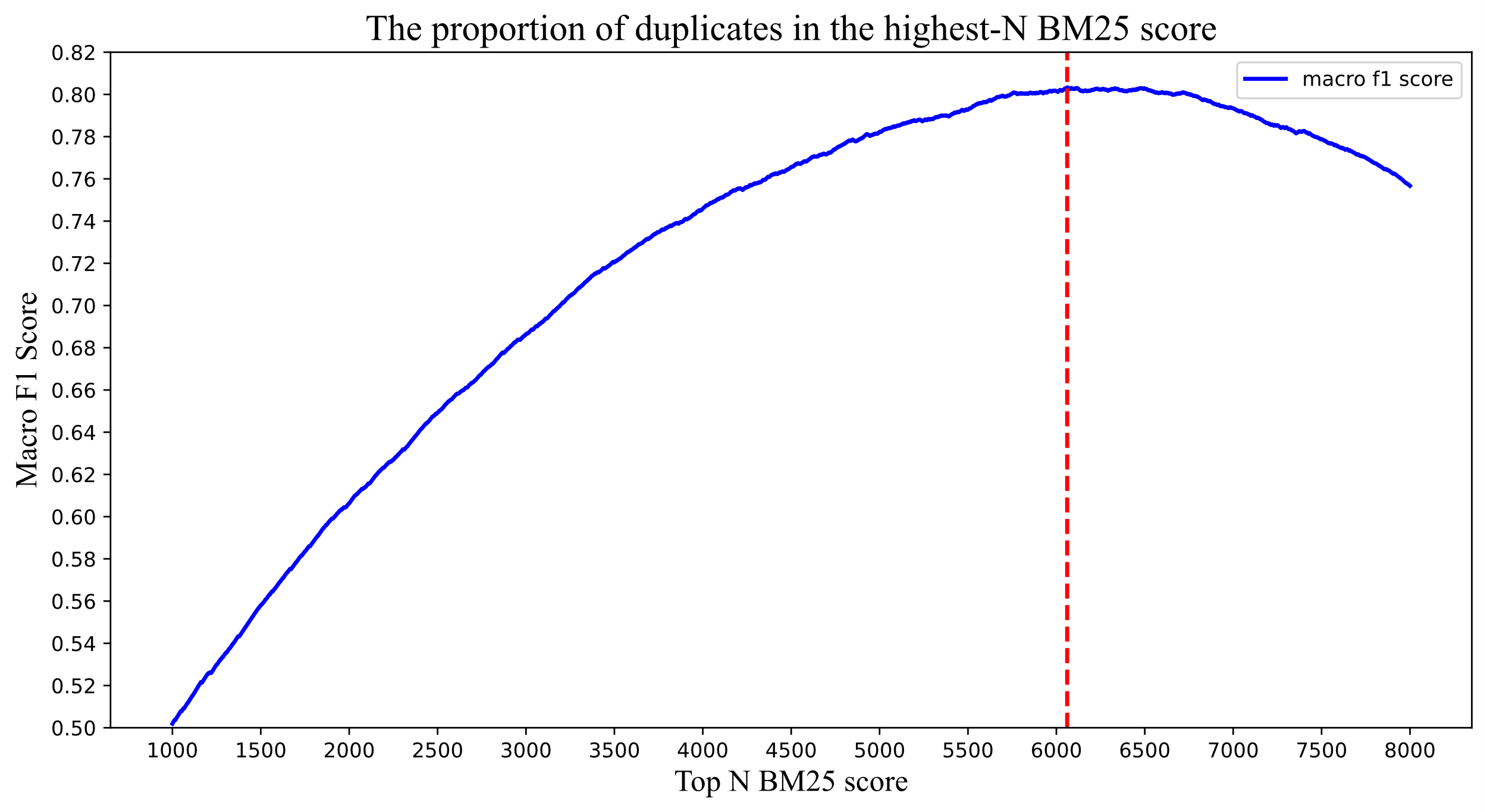


Fig. 7 Identification of the optimal BM25 score and the optimal rank.

Table 1. The selection criteria for climate-related policy data.

| Datasets | Selection basis | Items |
| --- | --- | --- |
| ECOLEX Legislation | Subjects | Agricultural & rural development, Air & atmosphere, Energy, Environment gen., Forestry, General, Land & soil, Mineral resources |

Notes: Some datasets such as “Overton” are not open-source and have relatively coarse classifications, hence they are not included in the scope of data sources for GCCMPD.

Table 2. Summary and comparison of IEA, CP, and CCLW.

| **Dataset** | **CCLW** | **CP** | **IEA** |
| --- | --- | --- | --- |
| Source | https://climate-laws.org/ | https://climatepolicydatabase.org/ | https://www.iea.org/policies |
| Institute | 1. the Grantham Research Institute on Climate Change 2. the Environment at the London School of Economics and Political Science 3. the Sabin Center on Climate Change Law at the Columbia Law School, New York | maintained by NewClimate Institute with support from PBL Netherlands Environmental Assessment Agency and Wageningen University and Research | IEA |
| Number | 2503 as of 2021.12.31 | 6536 as of 2021.12.31 | 5207 as of 2021.12.31 |
| Entity | 199 | 196 | 156 |
| Jurisdiction | Only national level | City, Country, Subnational region, Supranational region ***in Jurisdiction*** | National, State/Provincial, City/Municipal, Unknown, International, Regional ***in Jurisdiction*** |
| Mitigation or Adaptation | Adaptation, Disaster Risk Management, Loss and Damage, Mitigation ***in Responses*** | partial classification ***in Policy objective*** | No classification |
| Instrument | Capacity Building, Regulation, Incentives, Direct Investment, Governance and Planning (have Secondary classification) ***in Instruments***^[[8]](#footnote-8)^ | Barrier removal, Climate strategy, Economic instruments, Information and education, Policy support, Regulatory Instruments, Research & Development and Deployment (RD&D), Target, Voluntary approaches (have Secondary classification) ***in Type of policy instrument*** | Codes and standards, Regulation, Grants, Payments and transfers, Strategic plans, Information and education, Performance-based policies, Targets, plans and framework legislation, Payments, finance and taxation ***in Type*** |
| Sector | Agriculture, Transportation, Energy, Waste, Environment, Tourism, LULUCF, Industry, Residential and Commercial, Buildings, Water, Health, the Public Sector, and Other ***in Sectors*** | Agriculture and forestry, Buildings, Electricity and heat, General, Industry, Transport (have Secondary classification) ***in Sector name*** | Buildings, Residential, Economy-wide (Multisector), Industry, Transport, Road transport, Power generation, Services, Electricity and heat generation, Power, Heat and Utilities ***in Sector*** |
| Binding force | Very detailed and professional ***in Document Types*** | No classification | No classification |
| Executive/legislative^[[9]](#footnote-9)^ | Executive, Legislative ***in Type*** | A few ***in Policy description*** | No classification |
| Policy Content | Content has been summarized and condensed through more legal aspects ***in Description*** | Content is short (Extract only key information (such as Target)), many missing values (1813 of 5207, 496 only contain executive and legislative) ***in Policy description*** | Full content |
| Policy stringency | No | 1-5 (very few 105 of 5207) ***in Policy stringency*** | No |
| Objective | Such as Air Pollution, Biofuels, Biomass, Energy Efficiency  partial classification ***in Keywords*** | Such as Air Pollution ***in Policy objective***  Energy efficiency, Energy service demand reduction and resource efficiency, Nonenergy use, Other low-carbon technologies and fuel switch, Renewables ***in Policy type*** | Energy Efficiency, Air Quality, Digitalization, Electrification, Energy Access, Renewable Energy, Technology R&D and innovation, Methane abatement, Carbon Capture Utilization and Storage, Cities ***in Topics*** |
| Targets | Detailed targets for each country are provide. | Energy efficiency target, GHG reduction target, Renewable energy target (have Secondary classification) ***in Type of policy instrument*** | No |

Table 3. GCCMPD Sector Classification Reference.

| IPCC sector classification | Description | Subsector |
| --- | --- | --- |
| Energy systems | Public Electricity Generation | Electricity & heat |
| Energy systems | Public Combined Heat and Power gen. | Electricity & heat |
| Energy systems | Public Heat Plants | Electricity & heat |
| Energy systems | Public Electricity Generation (own use) | Electricity & heat |
| Energy systems | Electricity Generation (autoproducers) | Electricity & heat |
| Energy systems | Combined Heat and Power gen. (autoprod.) | Electricity & heat |
| Energy systems | Heat Plants (autoproducers) | Electricity & heat |
| Energy systems | Public Electricity Generation (biomass) | Biomass energy systems |
| Energy systems | Public Combined Heat and Power gen. (biom.) | Biomass energy systems |
| Energy systems | Public Heat Plants (biomass) | Biomass energy systems |
| Energy systems | Public Electricity Gen. (own use) (biom.) | Biomass energy systems |
| Energy systems | Electricity Generation (autoproducers) (biom.) | Biomass energy systems |
| Energy systems | Combined Heat and Power gen. (autopr.) (biom.) | Biomass energy systems |
| Energy systems | Heat Plants (autoproducers) (biomass) | Biomass energy systems |
| Energy systems | Refineries | Petroleum refining |
| Energy systems | Refineries (biomass) | Biomass energy systems |
| Energy systems | Gas works | Other incl. Indirect N2O |
| Energy systems | Other transformation sector (BKB, etc.) | Other incl. Indirect N2O |
| Energy systems | Gas works (biom.) | Biomass energy systems |
| Energy systems | Fuel comb. charcoal production (biom.) | Biomass energy systems |
| Energy systems | Other transf. sector (BKB, etc.) (biom.) | Biomass energy systems |
| Energy systems | Hard coal mining (gross) | Coal mining fugitive emissions |
| Energy systems | Methane recovery from coal mining | Coal mining fugitive emissions |
| Energy systems | Abandoned mines | Coal mining fugitive emissions |
| Energy systems | Brown coal mining | Coal mining fugitive emissions |
| Energy systems | Fuel transformation in gas works | Coal mining fugitive emissions |
| Energy systems | Fuel transformation charcoal production | Biomass energy systems |
| Energy systems | Fuel transformation of solid fuels (BKB Plants, coal liquefaction, patent fuel plants) | Coal mining fugitive emissions |
| Energy systems | Oil production | Oil and gas fugitive emissions |
| Energy systems | Oil production (biom.) | Biomass energy systems |
| Energy systems | Oil transmission | Oil and gas fugitive emissions |
| Energy systems | Tanker loading | Oil and gas fugitive emissions |
| Energy systems | Tanker oil transport (crude and NGL) | Oil and gas fugitive emissions |
| Energy systems | Transport by oil trucks | Oil and gas fugitive emissions |
| Energy systems | Oil refineries (evaporation) | Oil and gas fugitive emissions |
| Energy systems | Fuel transformation from liquid fuels (petrochemical plants) | Oil and gas fugitive emissions |
| Energy systems | Gas production | Oil and gas fugitive emissions |
| Energy systems | Gas transmission | Oil and gas fugitive emissions |
| Energy systems | Gas distribution | Oil and gas fugitive emissions |
| Energy systems | Fuel transformation of gaseous fuels (GTL, Blend, (re-)gasif./Liquef., NSF) | Oil and gas fugitive emissions |
| Energy systems | Venting and flaring during oil and gas production | Oil and gas fugitive emissions |
| Energy systems | Electrical Equipment Use (incl. site inst.) | Other incl. Indirect N2O |
| Energy systems | Coal fires (underground) | Other incl. Indirect N2O |
| Energy systems | Oil fires (Kuwait) | Other incl. Indirect N2O |
| Energy systems | Indirect N2O from NOx emitted in cat. 1A | Other incl. Indirect N2O |
| Energy systems | Indirect N2O from NH3 emitted in cat. 1A | Other incl. Indirect N2O |
| AFOLU | Agriculture and forestry (fos.) | Fuel combustion (REMOVE FROM FIGURES) |
| AFOLU | Agriculture and forestry (biom.) | Fuel combustion (REMOVE FROM FIGURES) |
| AFOLU | Off-road machinery: agric./for. (diesel) | Fuel combustion (REMOVE FROM FIGURES) |
| AFOLU | Fishing (fos.) | Fuel combustion (REMOVE FROM FIGURES) |
| AFOLU | Fishing (biom.) | Fuel combustion (REMOVE FROM FIGURES) |
| AFOLU | Non-specified other (fos.) | Fuel combustion (REMOVE FROM FIGURES) |
| AFOLU | Non-specified other (biom.) | Fuel combustion (REMOVE FROM FIGURES) |
| AFOLU | Dairy cattle | Enteric Fermentation (CH4) |
| AFOLU | Non-dairy cattle | Enteric Fermentation (CH4) |
| AFOLU | Buffalo | Enteric Fermentation (CH4) |
| AFOLU | Sheep | Enteric Fermentation (CH4) |
| AFOLU | Goats | Enteric Fermentation (CH4) |
| AFOLU | Camels and Lamas | Enteric Fermentation (CH4) |
| AFOLU | Horses | Enteric Fermentation (CH4) |
| AFOLU | Mules and asses | Enteric Fermentation (CH4) |
| AFOLU | Swine | Enteric Fermentation (CH4) |
| AFOLU | Manure Man.: Dairy Cattle (confined) | Manure management (N2O, CH4) |
| AFOLU | Manure Man.: Non-Dairy Cattle (confined) | Manure management (N2O, CH4) |
| AFOLU | Manure Man.: Buffalo (confined) | Manure management (N2O, CH4) |
| AFOLU | Manure Man.: Sheep (confined) | Manure management (N2O, CH4) |
| AFOLU | Manure Man.: Goats (confined) | Manure management (N2O, CH4) |
| AFOLU | Manure Man.: Camels and llamas (confined) | Manure management (N2O, CH4) |
| AFOLU | Manure Man.: Horses (confined) | Manure management (N2O, CH4) |
| AFOLU | Manure Man.: Mules and asses (confined) | Manure management (N2O, CH4) |
| AFOLU | Manure Man.: Swine (confined) | Manure management (N2O, CH4) |
| AFOLU | Manure Man.: Poultry (confined) | Manure management (N2O, CH4) |
| AFOLU | Rice cultivation (CH4) | Rice cultivation (CH4) |
| AFOLU | Synthetic Fertilizers | Synthetic fertilizer application (N2O) |
| AFOLU | Animal Manure Applied to Soils | Managed soils and pasture (CO2, N2O) |
| AFOLU | Direct soil emissions | Managed soils and pasture (CO2, N2O) |
| AFOLU | Crop Residue | Managed soils and pasture (CO2, N2O) |
| AFOLU | Cultivation of Histosols | Managed soils and pasture (CO2, N2O) |
| AFOLU | Pasture, Range and Paddock Manure | Managed soils and pasture (CO2, N2O) |
| AFOLU | Indirect N2O: Atm. Depos. - agricult. (4D) | Managed soils and pasture (CO2, N2O) |
| AFOLU | Indirect N2O: Leaching and Run-Off - agri. | Managed soils and pasture (CO2, N2O) |
| AFOLU | CO2 from urea application | Managed soils and pasture (CO2, N2O) |
| AFOLU | CO2 from agricultural lime application | Managed soils and pasture (CO2, N2O) |
| AFOLU | Savannah fires | Biomass burning (CO2, CH4) |
| AFOLU | Field burning of agric. res.: cereals | Biomass burning (CO2, CH4) |
| AFOLU | Field burning of agric. res.: pulses | Biomass burning (CO2, CH4) |
| AFOLU | Field burning of agric. res.: tuber and roots | Biomass burning (CO2, CH4) |
| AFOLU | Field burning of agric. res.: sugar cane | Biomass burning (CO2, CH4) |
| AFOLU | Field burning of agric. res.: other | Biomass burning (CO2, CH4) |
| Buildings | Commercial and public services (fos.) | Non-residential |
| Buildings | Commercial and public services (biom.) | Non-residential |
| Buildings | Residential (fos.) | Residential |
| Buildings | Residential (biom.) | Residential |
| Buildings | Fire Extinguishers | Non-CO2 (all buildings) |
| Buildings | Aerosols | Non-CO2 (all buildings) |
| Buildings | Adiabatic prop.: shoes and others | Non-CO2 (all buildings) |
| Buildings | Soundproof windows | Non-CO2 (all buildings) |
| Transport | Domestic air transport | Domestic Aviation |
| Transport | Road transport (incl. evap.) (foss.) | Road |
| Transport | Road transport (incl. evap.) (biom.) | Road |
| Transport | Non-road transport (rail, etc.) (fos.) | Rail |
| Transport | Non-road transport (rail, etc.)(biom.) | Rail |
| Transport | Inland shipping (fos.) | Inland Shipping |
| Transport | Inland shipping (biom.) | Inland Shipping |
| Transport | Non-road transport (fos.) | Other incl. Indirect N2O |
| Transport | Non-road transport (biom.) | Other incl. Indirect N2O |
| Transport | International air transport | International Aviation |
| Transport | International marine transport (bunkers) | International Shipping |
| Transport | International marine transport (biom.) | International Shipping |
| Transport | Adiabatic prop.: tyres | Road |
| Industry | Fuel combustion coke ovens | Metals |
| Industry | Blast furnaces (pig iron prod.) | Metals |
| Industry | Iron and steel | Metals |
| Industry | Iron and steel (biomass) | Metals |
| Industry | Non-ferrous metals | Metals |
| Industry | Non-ferrous metals (biomass) | Metals |
| Industry | Chemicals | Chemicals |
| Industry | Chemicals (biomass) | Chemicals |
| Industry | Pulp and paper | Other industry |
| Industry | Pulp and paper (biomass) | Other industry |
| Industry | Food and tobacco | Other industry |
| Industry | Food and tobacco (biomass) | Other industry |
| Industry | Other industries (stationary) (fos.) | Other industry |
| Industry | Off-road machinery: construction (diesel) | Other industry |
| Industry | Off-road machinery: mining (diesel) | Other industry |
| Industry | Other industries (stationary) (biom.) | Other industry |
| Industry | Off-road machinery: mining (diesel) | Other industry |
| Industry | Fuel transformation coke ovens | Metals |
| Industry | Cement production | Cement |
| Industry | Lime production | Chemicals |
| Industry | Limestone and Dolomite Use | Chemicals |
| Industry | Soda ash production | Chemicals |
| Industry | Soda ash use | Chemicals |
| Industry | Glass production | Chemicals |
| Industry | Ammonia production (gross CO2) | Chemicals |
| Industry | CO2-ammonia stored in urea | Chemicals |
| Industry | Nitric acid production | Chemicals |
| Industry | Adipic acid production | Chemicals |
| Industry | Silicon carbide production | Chemicals |
| Industry | Calcium carbide production | Chemicals |
| Industry | Carbon black production | Chemicals |
| Industry | Ethylene production | Chemicals |
| Industry | Styrene production | Chemicals |
| Industry | Methanol production | Chemicals |
| Industry | Caprolactam production | Chemicals |
| Industry | Other bulk chemicals production | Chemicals |
| Industry | Urea production | Chemicals |
| Industry | Vinyl chloride production | Chemicals |
| Industry | Glyoxal production | Chemicals |
| Industry | Crude steel production total | Metals |
| Industry | Blast furnaces | Metals |
| Industry | Sinter production | Metals |
| Industry | Ferroy Alloy production | Metals |
| Industry | Aluminium production (primary) | Metals |
| Industry | Aluminium production (secondary) | Metals |
| Industry | Magnesium foundries: SF6 use | Metals |
| Industry | Aluminium foundries: SF6 use | Metals |
| Industry | Lead production (primary) | Metals |
| Industry | Magnesium production (primary) | Metals |
| Industry | Zinc production (primary) | Metals |
| Industry | Production of halocarbons | Chemicals |
| Industry | Refrigeration and Air Conditioning | Other industry |
| Industry | Foam Blowing | Other industry |
| Industry | F-gas as Solvent | Other industry |
| Industry | Semiconductor Manufacture | Other industry |
| Industry | Flat Panel Display (FPD) Manufacture | Other industry |
| Industry | Photo Voltaic (PV) Cell Manufacture | Other industry |
| Industry | Electrical Equipment Manufacture | Other industry |
| Industry | F-gas/ODP consumption | Other industry |
| Industry | Accelerators/HEP | Other industry |
| Industry | Misc. (AWACS, other military and misc.) | Other industry |
| Industry | Unknown SF6 use | Other industry |
| Industry | Non-energy use of lubricants/waxes (CO2) | Other industry |
| Industry | Other Non-energy use of fuels (CO2 only) | Other industry |
| Industry | Solvents in paint | Chemicals |
| Industry | Degreasing and dry cleaning | Chemicals |
| Industry | Chemical products | Chemicals |
| Industry | Other product use | Chemicals |
| Industry | Use of N2O as anaesthesia | Chemicals |
| Industry | Use of N2O in aerosol spray cans | Chemicals |
| Industry | Managed waste disposal on land | Waste |
| Industry | Industrial wastewater | Waste |
| Industry | Domestic and commercial wastewater | Waste |
| Industry | Waste incineration - hazardous | Waste |
| Industry | Waste incineration - biogenic | Waste |
| Industry | Waste incineration - uncontrolled MSW burning | Waste |
| Industry | Waste incineration - other non-biogenic | Waste |
| Industry | Other waste | Waste |
| Industry | Indirect N2O from NOx emitted in cat. 2-3 | Other industry |
| Industry | Indirect N2O from NH3 emitted in cat. 2-3 | Other industry |

Note: This table is from the Supplementary of Lamb et al., (2021). It should be noted that unlike carbon emissions, it is very common for a climate policy to involve multiple sectors (e.g., policies involving the entire industrial chain, energy planning, etc.). We adopt a multilabel approach in which multiple sector labels are assigned when a policy involves multiple sectors (the same method is applied to instrument and objective), and a policy that affects the entire economy is labelled “Multisector”. If a policy cannot be classified into any specific sector and cannot be determined to be economy-wide, then the policy has no sector-specific characteristics.

Table 4. GCCMPD Instrument Classification Reference.

| Policy Instruments | Energy | Transport | Buildings | Industry | AFOLU |
| --- | --- | --- | --- | --- | --- |
| Economic Instruments- -Taxes (Carbon taxes may be economy-wide) | Carbon taxes (*e.g. CO₂ tax on offshore oil and gas*) | Fuel taxes (*e.g. Fuel Tax Reform*); Congestion charges, vehicle registration fees, road tolls (*e.g. Parking space management*); Vehicle taxes *(e.g. Environment-related tax on vehicle)* | Carbon and/or energy taxes (either sectoral or economy wide) *(e.g. Energy Tax Overhaul)* | Carbon tax or energy tax *(e.g. Industry carbon tax)*; Waste disposal taxes or charges *(e.g. Tax on Waste Disposal)* | Fertilizer or Nitrogen taxes to reduce nitrous oxide *(e.g. the Ecological Sales Tax)* |
| Economic Instruments- -Tradable Allowances (May be economy-wide) | Emissions trading *(e.g., EU Emissions Trading System Phase 3 (2013-2020))*; Emission credits under the Kyoto Protocol's Clean Development Mechanism (CDM) *(e.g., Linkage of Kyoto Protocol Project Mechanisms with EU Emissions Trading Scheme European Union (27) (2004))*; Tradable Green Certificates *(e.g., Green Electricity Market)* | Fuel and vehicle standards (*e.g.* *Revision of the EU Emission Trading System*) | Tradable certificates for energy efficiency improvements (white certificates) *(e.g. Emissions Trading Scheme)* | Emissions trading *(e.g. EU Emission Trading System (EU-ETS))*; Emission credits under CDM *(e.g. Alberta Energy Efficiency Carbon Offset Protocol)*; Tradable Green Certificates | Emission credis under CDM; Compliance schemes outside Kyoto protocol (national schemes) *(e.g. Federal Greenhouse Gas Offset System)*; Voluntary carbon markets |
| Economic Instruments- Subsidies | Fossil fuel subsidy removal *(e.g., Fuel price adjustments (subsidies removal))*; Feed-in-tariffs for renewable energy *(e.g., Feed-in tariff for solar PV installations)*; Capital subsidies and insurance for 1st generation Carbon Dioxide Capture and Storage (CCS) *(e.g., CO2 avoidance and use in raw material industries)* | Biofuel subsidies (*e.g.* *Biodiesel tax exemption*); Vehicle purchase subsidies (*e.g.* *Ecobonus: Subsidy for low-emission vehicles*); Feebates (*e.g. Bonus-Malus: vehicle CO2 bonus and penalty system*) | Subsidies or tax exemptions for investment in efficient buildings, retrofits and products *(e.g. Financial Incentives for Investment in Residential Renewable Generation and Residential Efficiency)*; Subsidized loans *(e.g. Interest-free loans for deep home retrofits)* | Subsidies (e.g., for energy audits) *(e.g. Management System of Intensive Energy Consumption)*; Fiscal incentives (e. g., for fuel switching) *(e.g. Fiscal incentives - exemptions for taxes and duties)* | Credit lines for low carbon agriculture, sustainable forestry. *(e.g. Low-Carbon Agriculture Plan)* |
| Regulatory Approaches | Efficiency or environmental performance standards *(e.g., Reduction of Carbon Dioxide Emissions from Coal-fired Generation of Electricity Regulations)*; Renewable Portfolio Standards for renewable energy *(e.g., Renewable Portfolio Standard (RPS))*; Equitable access to electricity grid *(e.g., Electricity Industry Law)*; Legal status of longterm CO2 storage *(e.g., Revised State Aid Guidelines for Environmental Protection)* | Fuel economy performance standards (*e.g.* *Fuel-efficiency standards for commercial heavy-duty vehicles (HDVs)*); Fuel quality standards (*e.g.* *EU Climate and Energy Package: Quality standards for fuels and biofuels*); GHG emission performance standards (*e.g. Vehicle Greenhouse Gas Emission Regulations*); Regulatory restrictions to encourage modal shifts (road to rail) (*e.g. National Plan for Recovery and Resilience - Component 3.2: transport modal shift*); Restriction on use of vehicles in certain areas (*e.g. Participation in International Car-Free Day for Cities (In town without my car!) & European Mobility Week*); Environmental capacity constraints on airports; Urban planning and zoning restrictions (*e.g. Low Emission Zone (LEZ)*) | Building codes and standards *(e.g. National Construction Code (NCC) Commercial Buildings)*; Equipment and appliance standards *(e.g. Greenhouse and Energy Minimum Standards)*; Mandates for energy retailers to assist customers invest in energy efficiency *(e.g. Retailer Energy Efficiency Scheme)* | Energy efficiency standards for equipment *(e.g. ecoENERGY Efficiency for Equipment Standards and Labelling)*; Energy management systems (also voluntary) *(e.g. Management System of Intensive Energy Consumption)*; Voluntary agreements (where bound by regulation) *(e.g. Voluntary energy saving agreement: electricity-intensive companies)*; Labelling and public procurement regulations *(e.g. Energy Conservation in Government)* | National policies to support REDD+ including monitoring, reporting and verification *(e.g. Law 11.284/2006, establishing the management of Public Forests, Brazilian Forest Service and National Fund for Forest Development)*; Forest law to reduce deforestation *(e.g. Revision of Forestry Law)*; Air and water pollution control GHG precursors *(e.g. The Agricultural Greenhouse Gases Program)*; Land-use planning and governance *(e.g. Climate Change Action Plan for Ministry of Land Management, Urban Planning and Construction 2015-2018)* |
| Information Programmes |  | Fuel labelling (*e.g.* *Fuel consumption labelling standard (ADR81/02) and fuel consumption label*); Vehicle efficiency labelling (*e.g.* Vehicle efficiency labelling) | Energy audits *(e.g. Energy Audit Programme)*; Labelling programmes *(e.g. National program for residential labelling)*; Energy advice programmes *(e.g. Advice and support for building professionals: The Facilitator network)* | Energy audits *(e.g. Energy Audit Programme)*; Benchmarking *(e.g. Energy Efficiency Benchmarking Covenant)*; Brokerage for industrial cooperation *(e.g. Green Conversion Package - Green Research Platform)* | Certification schemes for sustainable forest practices *(e.g. ISPO palm oil certification)*; Information policies to support REDD+ including monitoring, reporting and verification *(e.g. National Strategy For Reduced Emissions from Deforestation and Forest Degradation (REDD ))* |
| Government Provision of Public Goods or Services | Research and development *(e.g., Gas Turbine Research Project)*; Infrastructure expansion (district heating / cooling or common carrier) *(e.g., Light for Mexico Programme)* | Investment in transit and human powered transport (*e.g.* *Improving the infrastructure for using bicycles*); Investment in alternative fuel infrastructure (*e.g. EV and Alternative Fuel Infrastructure Deployment Initiative*); Low emission vehicle procurement (*e.g. New Zealand Government Procurement (NZGP) - EV fleet purchases*) | Public procurement of efficient buildings and appliances *(e.g. Energy Efficiency Program in Public Buildings - PROCEL EPP)* | Training and education *(e.g. Law 13.186 on the Policy for Education on Sustainable Consumption)*; Brokerage for industrial cooperation *(e.g. DOE fund to Small Businesses for Clean Energy R&D Projects)* | Protection of national, state, and local forests. *(e.g. Forests (Protection of Mangroves) Regulations)*; Investment in improvement and diffusion of innovative technologies in agriculture and forestry *(e.g. Law 25.080 for investments in cultivated forests)* |
| Voluntary Actions |  |  | Labelling programmes for efficient buildings *(e.g. Improving the energy and emissions performance of buildings)*; Product eco-labelling *(e.g. Energy-Efficiency Label (Energy Star))* | Voluntary agreements on energy targets or adoption of energy management systems, or resource efficiency *(e.g. Planned Voluntary Emissions Trading Scheme)* | Promotion of sustainability by developing standards and educational campaigns |

Table 5. GCCMPD Objective Classification Reference.

| Economic | Social | Environmental |
| --- | --- | --- |
| Energy security  Employment impact  New business opportunity / economic activity  Productivity/competitiveness  Technological spillover / innovation | Health impact  Energy/mobility access  (Fuel) Poverty alleviation  Food security  Impact on local conflicts  Safety/disaster resilience  Gender impact | Ecosystem impact (e.g. via air pollution)  Land-use competition  Water use/quality  Biodiversity conservation  Urban heat island effect  Resource/material use impact |

Table 6. GCCMPD Binding Force classification reference.

| **Hard & Soft Law** | **Classification** | **Secondary classification** | **Descriptions** |
| --- | --- | --- | --- |
| Hard Law | Constitution (legislative) |  | The Constitution is the fundamental/supreme law of a nation country. |
|  | Statutes/Legislation | International Law (-) | The international law is enshrined in conventions, treaties, and standards. Treaties and conventions are written agreements that states willingly sign and ratify, and therefore are obliged to follow. Such agreements, which are also called statutes or protocols, govern the mutual relations between states. |
|  |  | Law/Act (almost legislative) | The phrases Act and Law are so close that they are sometimes used interchangeably. |
|  |  | Decree/Order/Ordinance (almost executive) | Executive orders are legally binding directives to an executive agency by the president or other executive acting within his or her constitutional authority. Presidential decrees are similar to executive orders in that they are legally binding and issued by the president or executive. |
|  |  | Common Law/Case Law (executive) | Only a minority of countries accept the concept of common law, but where it is recognized, it is a source of authority in law. |
|  | Regulation/Directive/Decision (almost executive) |  | Regulations are issued under the authority of a statute by a division of the government or by a special body. The regulation, directive and decision are attributed legally binding force. |
| Soft Law & Quasilegislative | Preparatory and Informative Instruments  (prelaw function) | Preparatory Instruments (prelaw function) | Mainly include green papers, white papers, and action programs (general programs, action plans, agenda, etc.). Sometimes, action programmes with a legal basis such as Interim Measure are classified as regulation. |
|  |  | Informative Instruments (partially prelaw function) | Usually, include interinstitutional communications, purely informative communications, and individual communications. |
|  | Interpretative and Decisional Instruments  (postlaw function) | Interpretative Communications and Notices (postlaw function) | To elucidate the interpretation that should be given to the existing body of law, not to aim at laying down or creating new legal rules. |
|  |  | Decisional Notices and Communications (postlaw function) | Sometimes giving executive and discretionary powers is the main difference from interpretive instruments. Decisional notices do not aim at establishing new legal rules. |
|  |  | Decisional Guidelines, Codes and Frameworks (postlaw function) | Guidelines, codes, and frameworks further clarify the scope of the law and set out specific obligations. Usually, industry codes and guidelines, not legally binding. |
|  | Steering Instruments  (partially para-law function) |  | Very commonly used, mainly including recommendations, opinions, resolutions, codes of conduct, conclusions, and declarations. Refer exclusively to legal and/or political instruments, as opposed to economic and financial instruments. The primary objective of the instruments that fall within this category is to steer or guide action in a legally nonbinding way. |
|  | Other Strategy Plan or Target |  | The remaining strategies that cannot be judged (such as source documents cannot be found) or classified as hard law and the soft law classification above belong to this category. |

Note: Two points need to be pointed out. First, regarding the classification of hard law and soft law, we regard it as a single-label classification. Although some soft laws may have several functions, they will only appear between several similar soft laws. The classification will not affect the judgement of soft and hard laws, and this phenomenon is not common in our data. Second, the classification on legal hierarchy is a general standard and may vary slightly from country to country.

Table 7. GCCMPD Binding force Classification examples.

| Binding force Classification | Examples |
| --- | --- |
| Preparatory Instruments | Germany's Integrated National Energy and Climate Plan |
| Informative Instruments | USA’s Federal Wind Siting Information Center |
| Interpretative Communications and Notices | France’s New Ways to Calculate Engine Size |
| Decisional Notices and Communications | China’s Notice on Provisional Management Measures for Distributed Wind Power Project Development and Construction for all provinces |
| Decisional Guidelines, Codes and Frameworks | UK’s Code for Sustainable Homes |
| Steering Instruments | Mexico’s Resolution on met-metering) |

Table 8. Dictionary mapping of IEA Instruments and Sector-Instruments.

| **Keywords** | **Instrument** | **Sector-Instrument** |
| --- | --- | --- |
| Accelerated depreciation | Subsidies | |
| Associated pollutant limitations (SOx, VOCs, etc.) | Regulatory Approaches | Efficiency or environmental performance standards; Energy efficiency standards for equipment |
| Audits and inspections | Information Programmes | Energy audits; Energy audits; Information policies to support REDD+ including monitoring, reporting and verification |
| Awards | Government Provision of Public Goods or Services | |
| Building code (Prescriptive) | Regulatory Approaches | Building codes and standards |
| Building codes (performance-based) | Regulatory Approaches | Building codes and standards |
| Building codes and standards | Regulatory Approaches | Building codes and standards |
| Business accelerators / Incubators | Government Provision of Public Goods or Services | Brokerage for industrial cooperation |
| Business activity surveys | Government Provision of Public Goods or Services; Information Programmes | |
| Capacity auction | |  |
| Carbon tax | Taxes | Carbon taxes; Carbon and/or energy taxes; Carbon tax or energy tax |
| Climate change strategies | Government Provision of Public Goods or Services | |
| Co-funding via investment fund | Government Provision of Public Goods or Services | |
| Codes and standards | Regulatory Approaches | Energy efficiency standards for equipment |
| Company car tax | |  |
| Comparison labels | Information Programmes | |
| Compliance requirements | Regulatory Approaches | |
| Congestion charge | | |
| Contracts for difference | Government Provision of Public Goods or Services | |
| Consumer information | Information Programmes | |
| Education and training | Government Provision of Public Goods or Services | Training and education |
| Emission Trading Scheme | Tradable Allowances | Emissions trading; Fuel and vehicle standards; Tradable certificates for energy efficiency improvements (white certificates); Emissions trading; Emission credis under CDM |
| Emission standards | Regulatory Approaches | Efficiency or environmental performance standards; GHG emission performance standards; Energy efficiency standards for equipment; Air and water pollution control GHG precursors |
| Emissions estimates | Information Programmes | |
| Endorsement labels | Information Programmes | Fuel labelling; Labelling programmes; Certification schemes for sustainable forest practices |
| Energy / CO2 performance certification: Comparison | Information Programmes | Fuel labelling; Labelling programmes; Energy audits; Information policies to support REDD+ including monitoring, reporting and verification |
| Energy / CO2 performance labels | Information Programmes | Fuel labelling; Labelling programmes; Energy audits; Information policies to support REDD+ including monitoring, reporting and verification |
| Energy auction | |  |
| Energy efficiency / Fuel economy obligations | Regulatory Approaches | Efficiency or environmental performance standards; Fuel economy performance standards; Mandates for energy retailers to assist customers invest in energy efficiency; Energy efficiency standards for equipment |
| Energy market regulation | Regulatory Approaches | |
| Energy trading regulations | Regulatory Approaches | |
| Enforcement | Regulatory Approaches | |
| Environmental Impact Assessment | Regulatory Approaches; Information Programmes | |
| Equipment sales obligation | Regulatory Approaches | Efficiency or environmental performance standards; Equipment and appliance standards; Energy efficiency standards for equipment |
| Equity |  |  |
| Excise taxes | |  |
| Externality taxation | | |
| Feebate | Subsidies | Feebates |
| Feed-in tariffs/premiums | Subsidies | Feed-in-tariffs for renewable energy |
| Finance | Government Provision of Public Goods or Services | |
| Framework legislation | Government Provision of Public Goods or Services | |
| Fuel quality standards | Regulatory Approaches | Fuel quality standards |
| Funds to sub-national governments | Government Provision of Public Goods or Services | |
| GHG taxation | |  |
| GHG emissions liability | Regulatory Approaches | |
| Government provided advice | Information Programmes | Energy advice programmes |
| Grants | Government Provision of Public Goods or Services | |
| Import tax | |  |
| Inducement prizes | Government Provision of Public Goods or Services | |
| Industrial clusters | Government Provision of Public Goods or Services | Brokerage for industrial cooperation |
| Information and education | Government Provision of Public Goods or Services; Information Programmes | |
| Information campaigns | Information Programmes | |
| Insurance | Subsidies | Capital subsidies and insurance for 1st generation Carbon Dioxide Capture and Storage (CCS) |
| Intellectual property regimes | Regulatory Approaches | |
| International collaboration | Government Provision of Public Goods or Services | |
| Investment in start-ups | Government Provision of Public Goods or Services | |
| Investment in assets | Government Provision of Public Goods or Services | Infrastructure expansion (district heating / cooling or common carrier); Investment in alternative fuel infrastructure; Public procurement of efficient buildings and appliances; Investment in improvement and diffusion of innovative technologies in agriculture and forestry |
| Investment tax incentives | Subsidies | Fiscal incentives |
| Knowledge networks | Government Provision of Public Goods or Services; Information Programmes | Research and development; Investment in improvement and diffusion of innovative technologies in agriculture and forestry |
| Knowledge sharing | Government Provision of Public Goods or Services; Information Programmes | Research and development; Investment in improvement and diffusion of innovative technologies in agriculture and forestry |
| Knowledge sharing requirements | Government Provision of Public Goods or Services; Information Programmes; Regulatory Approaches | Research and development; Investment in improvement and diffusion of innovative technologies in agriculture and forestry |
| Leak detection and repair requirements | Regulatory Approaches | |
| Loan guarantee | Subsidies | Subsidized loans; Credit lines for low carbon agriculture, sustainable forestry |
| Loans (incl. concessional loans) | Subsidies | Subsidized loans; Credit lines for low carbon agriculture, sustainable forestry |
| Loans / debt finance | Subsidies | Subsidized loans; Credit lines for low carbon agriculture, sustainable forestry |
| Long-term low emissions development strategy (LT-LEDS) | Government Provision of Public Goods or Services | |
| Luxury tax | |  |
| Major infrastructure plan | Government Provision of Public Goods or Services | Infrastructure expansion (district heating / cooling or common carrier); Investment in alternative fuel infrastructure; Public procurement of efficient buildings and appliances |
| Mandatory energy management system | Regulatory Approaches | Energy management systems |
| Mandatory reporting | Information Programmes; Regulatory Approaches | Fuel labelling; Energy audits; Energy audits; Information policies to support REDD+ including monitoring, reporting and verification |
| Mandatory technology use | Regulatory Approaches | Efficiency or environmental performance standards; Fuel quality standards; Energy efficiency standards for equipment |
| Market design rules | Regulatory Approaches | |
| Matchmaking between investors and firms | Government Provision of Public Goods or Services | Brokerage for industrial cooperation |
| Measurement, calibration, equipment requirements | Regulatory Approaches | Equipment and appliance standards; Energy efficiency standards for equipment |
| Metering and connection requirements | Regulatory Approaches | |
| Minimum energy performance standards | Regulatory Approaches | Efficiency or environmental performance standards; Fuel economy performance standards; Equipment and appliance standards; Energy efficiency standards for equipment |
| Monitoring | Information Programmes | Energy audits; Energy audits; Information policies to support REDD+ including monitoring, reporting and verification |
| National climate change strategy | | |
| Nationally Determined Contribution | | |
| Negotiated agreements (public-private sector) | Voluntary Actions | |
| Notice requirements | Information Programmes; Regulatory Approaches | |
| Obligations on average types of sales / output | Regulatory Approaches | |
| On-bill finance | |  |
| Operational funding for institutions | Government Provision of Public Goods or Services | |
| Other polluant liabilities | Regulatory Approaches | |
| Other regulatory instruments | Regulatory Approaches | |
| Parking charges | |  |
| Payments |  |  |
| Payments and transfers | | |
| Payments, finance and taxation | | |
| Peer-to-peer trading rules | Regulatory Approaches | |
| Performance-based payments | | |
| Performance-based policies | Information Programmes | |
| Permitting processes | Regulatory Approaches | |
| Pollution liability | Regulatory Approaches | |
| Pollution rights | Regulatory Approaches | |
| Prescriptive requirements and standards | Regulatory Approaches | Efficiency or environmental performance standards; Equipment and appliance standards; Energy efficiency standards for equipment |
| Prevenative maintenance requirements | Regulatory Approaches | |
| Primary / Secondary education | Government Provision of Public Goods or Services | Research and development; Training and education |
| Price controls (incl. social tariffs) | | |
| Procedural requirements | Regulatory Approaches | |
| Product certification | Information Programmes | Fuel labelling; Energy audits; Energy audits; Information policies to support REDD+ including monitoring, reporting and verification |
| Product taxation | | |
| Product-based MEPS | Regulatory Approaches | Efficiency or environmental performance standards; Fuel economy performance standards; Equipment and appliance standards; Energy efficiency standards for equipment |
| Product import or sales bans | Regulatory Approaches | |
| Professional / Vocational training and certification | Information Programmes; Government Provision of Public Goods or Services | Research and development; Training and education |
| Prohibition | Regulatory Approaches | |
| Public disclosure requirements | Information Programmes; Regulatory Approaches | Fuel labelling; Energy audits; Labelling and public procurement regulations; Energy audits; Information policies to support REDD+ including monitoring, reporting and verification |
| Public information | Information Programmes | Fuel labelling; Energy audits; Energy audits; Information policies to support REDD+ including monitoring, reporting and verification |
| Public procurement | Government Provision of Public Goods or Services | Infrastructure expansion (district heating / cooling or common carrier); Low emission vehicle procurement; Public procurement of efficient buildings and appliances |
| Public voluntary programmes | Voluntary Actions | Voluntary agreements on energy targets or adoption of energy management systems, or resource efficiency; Promotion of sustainability by developing standards and educational campaigns |
| Rebates | Subsidies | |
| Recordkeeping requirements | Information Programmes; Regulatory Approaches | |
| Regulation | Regulatory Approaches | |
| Renewable / Non-fossil energy obligations | Regulatory Approaches | Renewable Portfolio Standards for renewable energy; Fuel quality standards; Building codes and standards; Energy efficiency standards for equipment |
| Reporting | Information Programmes | |
| Resource rights | Regulatory Approaches | |
| Resource extraction taxes and royalties | | |
| Rights | Regulatory Approaches | |
| Rights, permits and licenses | Regulatory Approaches; Information Programmes | |
| Risk sharing facilities | Government Provision of Public Goods or Services | |
| Road usage charges | | |
| Safety standards | Regulatory Approaches | Efficiency or environmental performance standards; Fuel quality standards; Building codes and standards; Energy efficiency standards for equipment |
| Sectoral standards | Regulatory Approaches | Efficiency or environmental performance standards; Building codes and standards; Energy efficiency standards for equipment |
| Standards and laws for Green Bonds | Government Provision of Public Goods or Services | |
| Strategic plans | | |
| Sustainable finance frameworks | Government Provision of Public Goods or Services | |
| Targets |  |  |
| Targets, plans and framework legislation | Government Provision of Public Goods or Services | |
| Tariff design | Subsidies | Feed-in-tariffs for renewable energy |
| Tax credits and exemptions | Subsidies | Subsidies or tax exemptions for investment in efficient buildings, retrofits and products; Credit lines for low carbon agriculture, sustainable forestry. |
| Taxes, fees and charges | | |
| Technology bans / phase outs | Regulatory Approaches | |
| Technology roadmaps | Government Provision of Public Goods or Services | |
| Technology testing method | Information Programmes | Fuel labelling; Energy audits; Energy audits; Information policies to support REDD+ including monitoring, reporting and verification |
| Third party verification | Information Programmes | Fuel labelling; Energy audits; Energy audits; Information policies to support REDD+ including monitoring, reporting and verification |
| Time-of-use tariffs | Subsidies | Feed-in-tariffs for renewable energy |
| Unilateral commitments (private sector) | Voluntary Actions | |
| Urban planning | Government Provision of Public Goods or Services | |
| Use / activity restrictions | Regulatory Approaches | |
| Use and activity charges | | |
| Value added tax | Subsidies | |
| Vehicle registration tax | | |
| Voluntary approaches | Voluntary Actions | |
| Voluntary reporting | Voluntary Actions; Information Programmes | |
| finance and taxation | Government Provision of Public Goods or Services | |
| permits and licenses | Regulatory Approaches; Information Programmes | |
| plans and framework legislation | Government Provision of Public Goods or Services | |

Table 9. Dictionary mapping of Climate Policy Instruments and Sector-Instruments.

| **Keywords** | **Instrument** | **Sector-Instrument** |
| --- | --- | --- |
| Barrier removal | |  |
| Grid access and priority for renewables | Regulatory Approaches | Equitable access to electricity grid |
| Net metering | Subsidies | |
| Removal of fossil fuel subsidies | Subsidies | Fossil fuel subsidy removal |
| Removal of split incentives (landlord tenant problem) | Subsidies | |
| Climate strategy | |  |
| Coordinating body for climate strategy | Government Provision of Public Goods or Services | |
| Formal & legally binding climate strategy | | |
| Political & non-binding climate strategy | | |
| Economic instruments | | |
| Direct investment | Government Provision of Public Goods or Services | |
| Funds to sub-national governments | Government Provision of Public Goods or Services | |
| Infrastructure investments | Government Provision of Public Goods or Services | Infrastructure expansion (district heating / cooling or common carrier); Investment in alternative fuel infrastructure; Public procurement of efficient buildings and appliances; Investment in improvement and diffusion of innovative technologies in agriculture and forestry |
| Procurement rules | Government Provision of Public Goods or Services; Regulatory Approaches | Infrastructure expansion (district heating / cooling or common carrier); Low emission vehicle procurement; Public procurement of efficient buildings and appliances; Investment in improvement and diffusion of innovative technologies in agriculture and forestry |
| RD&D funding | Government Provision of Public Goods or Services | Research and development |
| Fiscal or financial incentives | | |
| CO2 taxes | Taxes | Carbon taxes; Fuel taxes; Carbon and/or energy taxes; Carbon tax or energy tax |
| Energy and other taxes | Taxes | Carbon taxes; Fuel taxes; Carbon and/or energy taxes; Carbon tax or energy tax; Fertilizer or Nitrogen taxes to reduce nitrous oxide |
| Feed-in tariffs or premiums | Subsidies | Feed-in-tariffs for renewable energy |
| Grants and subsidies | Government Provision of Public Goods or Services; Subsidies | |
| Loans | Subsidies | Subsidized loans; Credit lines for low carbon agriculture, sustainable forestry |
| Retirement premium | Subsidies | |
| Tax relief | Subsidies | |
| Tendering schemes | | |
| User charges | Subsidies | |
| Market-based instruments | | |
| GHG emission reduction crediting and offsetting mechanism | Tradable Allowances | Fuel and vehicle standards; Tradable certificates for energy efficiency improvements (white certificates) |
| GHG emissions allowances | Tradable Allowances | Emissions trading; Fuel and vehicle standards; Tradable certificates for energy efficiency improvements (white certificates); Emissions trading; Emission credis under CDM |
| Green certificates | Tradable Allowances | Tradable Green Certificates; Tradable Green Certificates |
| White certificates | Tradable Allowances | Tradable certificates for energy efficiency improvements (white certificates) |
| Information and education | | |
| Advice or aid in implementation | Information Programmes | Energy advice programmes |
| Information provision | Information Programmes | |
| Performance label | Information Programmes | Vehicle efficiency labelling; Labelling programmes |
| Comparison label | Information Programmes | Fuel labelling; Labelling programmes |
| Endorsement label | Information Programmes | Fuel labelling; Labelling programmes |
| Professional training and qualification | Government Provision of Public Goods or Services; Information Programmes | Training and education |
| Policy support | |  |
| Institutional creation | Government Provision of Public Goods or Services | |
| Strategic planning | Government Provision of Public Goods or Services | |
| Regulatory Instruments | | |
| Auditing | Information Programmes | Energy audits; Energy audits; Information policies to support REDD+ including monitoring, reporting and verification |
| Codes and standards | Regulatory Approaches | Efficiency or environmental performance standards; GHG emission performance standards; Building codes and standards; Energy efficiency standards for equipment; Air and water pollution control GHG precursors |
| Building codes and standards | Regulatory Approaches | Building codes and standards |
| Industrial air pollution standards | Regulatory Approaches | Efficiency or environmental performance standards; GHG emission performance standards; Energy efficiency standards for equipment; Air and water pollution control GHG precursors |
| Product standards | Regulatory Approaches | Efficiency or environmental performance standards; Fuel quality standards; Equipment and appliance standards; Energy efficiency standards for equipment; Air and water pollution control GHG precursors |
| Sectoral standards | Information Programmes; Regulatory Approaches | Efficiency or environmental performance standards; GHG emission performance standards; Building codes and standards; Energy efficiency standards for equipment; Air and water pollution control GHG precursors |
| Vehicle air pollution standards | Regulatory Approaches | GHG emission performance standards |
| Vehicle fuel-economy and emissions standards | Regulatory Approaches | Fuel economy performance standards; GHG emission performance standards |
| Monitoring | Information Programmes | Energy audits; Energy audits; Information policies to support REDD+ including monitoring, reporting and verification |
| Obligation schemes | Regulatory Approaches | |
| Other mandatory requirements | Regulatory Approaches | |
| Research & Development and Deployment (RD&D) | Government Provision of Public Goods or Services | Research and development; Investment in improvement and diffusion of innovative technologies in agriculture and forestry |
| Demonstration project | Government Provision of Public Goods or Services | Research and development; Investment in improvement and diffusion of innovative technologies in agriculture and forestry |
| Research programme | Government Provision of Public Goods or Services | Research and development; Investment in improvement and diffusion of innovative technologies in agriculture and forestry |
| Technology deployment and diffusion | Government Provision of Public Goods or Services | Research and development; Investment in improvement and diffusion of innovative technologies in agriculture and forestry |
| Technology development | Government Provision of Public Goods or Services | Research and development; Investment in improvement and diffusion of innovative technologies in agriculture and forestry |
| Target |  |  |
| Energy efficiency target | | |
| Formal & legally binding energy efficiency target | | |
| Political & non-binding energy efficiency target | | |
| GHG reduction target | | |
| Formal & legally binding GHG reduction target | | |
| Political & non-binding GHG reduction target | | |
| Renewable energy target | | |
| Formal & legally binding renewable energy target | | |
| Political & non-binding renewable energy target | | |
| Voluntary approaches | Voluntary Actions | |
| Negotiated agreements (public-private sector) | Voluntary Actions | |
| Public voluntary schemes | Voluntary Actions | |
| Unilateral commitments (private sector) | Voluntary Actions | |

Table 10. Dictionary mapping of CCLW Instruments and Sector-Instruments.

| **Keywords** | **Instrument** | **Sector-Instrument** |
| --- | --- | --- |
| Building codes\|Regulation | Regulatory Approaches | Building codes and standards |
| Capacity-building - general\|Capacity-building | Government Provision of Public Goods or Services | Research and development; Training and education; Investment in improvement and diffusion of innovative technologies in agriculture and forestry |
| Climate fund\|Governance and planning | Government Provision of Public Goods or Services | |
| Creating bodies and institutions\|Governance and planning | Government Provision of Public Goods or Services | |
| Designing processes\|Governance and planning | Government Provision of Public Goods or Services | |
| Developing plans and strategies\|Governance and planning | Government Provision of Public Goods or Services | |
| Disclosure obligations\|Regulation | Information Programmes; Regulatory Approaches | Fuel labelling; Energy audits; Energy audits; Information policies to support REDD+ including monitoring, reporting and verification |
| Ecosystem restoration and nature based solutions\|Direct investment | Government Provision of Public Goods or Services | Protection of national, state, and local forests |
| Education and training\|Capacity-building | Government Provision of Public Goods or Services | Training and education |
| Governance and Planning\|Creating bodies/institutions | Government Provision of Public Goods or Services | |
| Governance and Planning\|Developing processes | Government Provision of Public Goods or Services | |
| Governance and Planning\|MRV | Information Programmes | Fuel labelling; Energy audits; Energy audits; Information policies to support REDD+ including monitoring, reporting and verification |
| Governance and Planning\|Monitoring, Reporting, and Verification | Information Programmes | Energy audits; Energy audits; Information policies to support REDD+ including monitoring, reporting and verification |
| Incentives\|Other | Subsidies | Fiscal incentives |
| Insurance\|Incentives | Subsidies | |
| International cooperation\|Governance and planning | Government Provision of Public Goods or Services | |
| Knowledge generation\|Capacity-building | Government Provision of Public Goods or Services | Research and development; Investment in improvement and diffusion of innovative technologies in agriculture and forestry |
| Knowledge sharing and dissemination\|Capacity-building | Government Provision of Public Goods or Services; Information Programmes | Research and development; Investment in improvement and diffusion of innovative technologies in agriculture and forestry |
| Monitoring and evaluation\|Governance and planning | Information Programmes | Fuel labelling; Energy audits; Energy audits; Information policies to support REDD+ including monitoring, reporting and verification |
| Monitoring, Reporting, Verification\|Governance and planning | Information Programmes | Fuel labelling; Energy audits; Energy audits; Information policies to support REDD+ including monitoring, reporting and verification |
| Multi-level governance\|Governance and planning | Government Provision of Public Goods or Services | |
| Provision of climate finance\|Direct investment | Government Provision of Public Goods or Services | |
| Public goods - early warning systems\|Direct investment | Government Provision of Public Goods or Services | |
| Public goods - other\|Direct investment | Government Provision of Public Goods or Services | |
| Regulation\|Standards & obligations | Regulatory Approaches | Efficiency or environmental performance standards; GHG emission performance standards; Building codes and standards; Energy efficiency standards for equipment; Air and water pollution control GHG precursors |
| Research and development\|Capacity-building | Government Provision of Public Goods or Services | Research and development; Investment in improvement and diffusion of innovative technologies in agriculture and forestry |
| Social safety nets\|Direct investment | Government Provision of Public Goods or Services | |
| Standards and obligations\|Regulation | Regulatory Approaches | Efficiency or environmental performance standards; GHG emission performance standards; Building codes and standards; Energy efficiency standards for equipment; Air and water pollution control GHG precursors |
| Subsidies\|Incentives | Subsidies | Fiscal incentives |
| Taxes\|Incentives | Subsidies | Fiscal incentives |
| Zoning and spatial planning\|Regulation | Regulatory Approaches | |

Table 11. Dictionary mapping of IEA Sector and Subsector.

| **Keywords** | **Sector** | **Subsector** |
| --- | --- | --- |
| Economy-wide (Multi-sector) | Multi-sector | |
| Accomodation and food services | Buildings | Non-residential |
| Administration and offices | Buildings | Non-residential |
| Agriculture | AFOLU |  |
| Agriculture, Fisheries, Forestry and Hunting | AFOLU |  |
| Air transport | Transport | Domestic Aviation |
| Ammonia | Industry | Chemicals |
| Appartment in high-rise building | Buildings | Residential |
| Appartment in low-rise building | Buildings | Residential |
| Attached house | Buildings | Residential |
| Biodiesel | Transport | |
| Bioethanol | Transport | |
| Biofuel production | Transport | |
| Buildings | Buildings | |
| Buses - Rapid transit and intercity service | Transport | Road |
| Buses and minibuses - Local and urban service | Transport | Road |
| Chemical and petrochemicals | Industry | Chemicals |
| Coal and lignite mining | Energy systems | Coal mining fugitive emissions |
| Coal secondary products production | Energy systems | Coal mining fugitive emissions |
| Combined heat and power | Energy systems | Electricity & heat |
| Construction | Buildings | |
| Cooling production and distribution (incl. district cooling) | Energy systems | Electricity & heat |
| CO2 capture | |  |
| CO2 transport, utilisation and storage | | |
| Data centre | |  |
| Delivery freight (Road) | Transport | Road |
| Demand response | |  |
| Detached house | Buildings | Residential |
| Distribution | |  |
| Domestic freight (Water) | Transport | Inland Shipping |
| Domestic passenger (Air) | Transport | Domestic Aviation |
| Downstream | |  |
| Education | |  |
| Electricity and heat generation | Energy systems | Electricity & heat |
| Electricity distribution | Energy systems | Electricity & heat |
| Electricity transmission | Energy systems | Electricity & heat |
| Existing buildings and retrofits | Buildings | |
| Exploration, drilling, well development and extraction | Industry | Other industry |
| Fisheries | AFOLU |  |
| Food and tobacco | Industry | Other industry |
| Food retail | Industry |  |
| Forestry | AFOLU |  |
| Forestry and Hunting | AFOLU |  |
| Fossil fuel production | Energy systems | Oil and gas fugitive emissions |
| Freight transport (Air) | Transport | Domestic Aviation |
| Freight transport (Rail) | Transport | Rail |
| Freight transport (Road) | Transport | Road |
| Freight transport (Water) | Transport | Inland Shipping |
| Fuel gathering and pre-refining processing (including bitumen upgrading) | Energy systems | Oil and gas fugitive emissions |
| Fuel processing and transformation | Energy systems | Oil and gas fugitive emissions |
| Health and social activities | | |
| Heat and Utilities | Energy systems | Electricity & heat |
| Heat and steam distribution (incl. district heating) | Energy systems | Electricity & heat |
| Heat generation | Energy systems | Electricity & heat |
| Heating and Cooling | Buildings | |
| High value chemicals | Industry | Chemicals |
| Hydrogen production | Multi-sector | |
| Hydrogen production and supply | Multi-sector | |
| Hydrogen storage | Multi-sector | |
| Hydrogen transportation | Energy systems | Oil and gas fugitive emissions |
| Industry | Industry |  |
| Intercity rail | Transport | Rail |
| International freight (Water) | Transport | International Shipping |
| International freight (Air) | Transport | International Aviation |
| International passenger (Air) | Transport | International Aviation |
| Investment in start-ups | | |
| Iron and steel | Industry | Metals |
| LNG transportation | Energy systems | Oil and gas fugitive emissions |
| Liquefaction | Energy systems | Coal mining fugitive emissions |
| Machinery | Industry | Other industry |
| Manufacturing | Industry |  |
| Mass road transit | Transport | Road |
| Metal ore mining | Industry | Metals |
| Methanol | Industry | Chemicals |
| Mining and quarrying (incl. fossil fuel extraction) | Energy systems | Oil and gas fugitive emissions |
| Multipurpose | |  |
| Natural gas | Energy systems | Oil and gas fugitive emissions |
| Natural gas processing | Energy systems | Oil and gas fugitive emissions |
| New buildings | Buildings | |
| Offshore |  |  |
| Oil and natural gas extraction | Energy systems | Oil and gas fugitive emissions |
| Oil and natural gas secondary products production | Energy systems | Oil and gas fugitive emissions |
| Onshore - Conventional | | |
| Onshore - Unconventional | | |
| Other light manufacturing | Industry | Other industry |
| Paper, pulp and printing | Industry | Other industry |
| Passenger transport (Air) | Transport | Domestic Aviation |
| Passenger transport (Rail) | Transport | Rail |
| Passenger transport (Road) | Transport | Road |
| Pipeline transportation | Energy systems | Oil and gas fugitive emissions |
| Plant-based | Energy systems | Electricity & heat |
| Power | Energy systems | Electricity & heat |
| Power generation | Energy systems | Electricity & heat |
| Power storage | Energy systems | Electricity & heat |
| Power transmission and distribution | Energy systems | Electricity & heat |
| Power, Heat and Utilities | Energy systems | Electricity & heat |
| Private - Individual (Road) | Transport | Road |
| Private transport companies (incl. taxis and VTCs) | Transport | Road |
| Processing | Energy systems | Oil and gas fugitive emissions |
| Public administration | Buildings | Non-residential |
| Public assembly | Buildings | Non-residential |
| Rail transport | Transport | Rail |
| Refining | Energy systems | Petroleum refining |
| Regasification | Energy systems | Oil and gas fugitive emissions |
| Repair | Buildings | |
| Repair, industrial and other service activities | Buildings | |
| Residential | Buildings | Residential |
| Restaurants | Buildings | Non-residential |
| Road transport | Transport | Road |
| SMEs |  |  |
| Services | Buildings | Non-residential |
| Sewerage, waste and remediation | Industry | Waste |
| Storage |  |  |
| Textile and leather | Industry | Other industry |
| Transmission | Energy systems | Oil and gas fugitive emissions |
| Transport | Transport | |
| Transport equipment | Transport | |
| Upstream |  |  |
| Urban and suburban rail | Transport | Rail |
| Water supply | |  |
| Water transport | Transport | Inland Shipping |
| Wholesale and retail | | |
| industrial and other service activities | Buildings | |

Table 12. Dictionary mapping of Climate Policy Sector and Subsector.

| **Keywords** | **Sector** | **Subsector** |
| --- | --- | --- |
| Agriculture and forestry | AFOLU |  |
| Agricultural CH4 | AFOLU |  |
| Agricultural CO2 | AFOLU |  |
| Agricultural N2O | AFOLU |  |
| Forestry | AFOLU |  |
| Buildings | Buildings | |
| Appliances | Buildings | Residential |
| Construction | Buildings | |
| Heating and cooling | Buildings | |
| Hot water and cooking | Buildings | Residential |
| Electricity and heat | Energy systems | Electricity & heat |
| CCS | Energy systems | Electricity & heat |
| Coal | Energy systems | Electricity & heat |
| Gas | Energy systems | Electricity & heat |
| Nuclear | Energy systems | Electricity & heat |
| Oil | Energy systems | Electricity & heat |
| Renewables | Energy systems | Electricity & heat |
| General | Multi-sector | |
| Industry | Industry |  |
| Fluorinated gases | Industry | Other industry |
| Fossil fuel exploration and production | Energy systems | Oil and gas fugitive emissions |
| Industrial energy related | Industry |  |
| Industrial N2O | Industry |  |
| Industrial process CO2 | Industry |  |
| Negative emissions | Industry |  |
| Waste CH4 | Industry | Waste |
| Transport | Transport | |
| Air | Transport | Domestic Aviation |
| Heavy-duty vehicles | Transport | Road |
| Light-duty vehicles | Transport | Road |
| Low-emissions mobility | Transport | Road |
| Rail | Transport | Rail |
| Shipping | Transport | Inland Shipping |

Table 13. Dictionary mapping of CCLW Sector and Subsector.

| **Keywords** | **Sector** | **Subsector** |
| --- | --- | --- |
| Economy-wide | Multi-sector | |
| Transport | Transport | |
| Tourism |  |  |
| Transportation | Transport | |
| Industry | Industry |  |
| Waste | Industry | Waste |
| Water |  |  |
| Energy | Energy systems | |
| Residential and Commercial | Buildings | Residential; Non-residential |
| Rural |  |  |
| Urban |  |  |
| Buildings | Buildings | |
| Coastal zones | |  |
| Cross Cutting Area | | |
| LULUCF | AFOLU |  |
| Agriculture | AFOLU |  |
| Health |  |  |
| Social development | | |
| Environment | |  |
| Finance |  |  |
| Disaster Risk Management (Drm) | | |
| Adaptation | |  |
| Public Sector | |  |
| Other |  |  |

Table 14. Dictionary mapping of IEA Objective and Subobjective.

| **Keywords** | **Objective** | **Subobjective** |
| --- | --- | --- |
| Air Quality | Social; Environmental | Health impact; Ecosystem impact |
| Carbon Capture Utilisation and Storage | Environmental | Ecosystem impact |
| Cities | Social |  |
| Critical Minerals | Environmental | Resource/material use impact |
| Digitalisation | Social | Energy/mobility access |
| Electrification | Social | Energy/mobility access |
| Energy Access | Social | Energy/mobility access |
| Energy Efficiency | Economic | Productivity/competitiveness |
| Energy Poverty | Social | (Fuel) Poverty alleviation |
| Energy Security | Economic | Energy security |
| Energy Water Nexus | Environmental | Water use/quality |
| Methane abatement | Environmental | Ecosystem impact |
| Renewable Energy | |  |
| Technology R&D and innovation | Economic | Technological spillover/innovation |

Table 15. Dictionary mapping of Climate Policy Objective and Subobjective.

| **Keywords** | **Objective** | **Subobjective** |
| --- | --- | --- |
| Adaptation | |  |
| Air pollution | Social; Environmental | Health impact; Ecosystem impact |
| Economic development | Economic |  |
| Energy access | Social | Energy/mobility access |
| Energy security | Economic | Energy security |
| Food security | Social | Health impact; Food security |
| Land use | Environmental | Land-use competition |
| Mitigation | |  |
| Water | Social; Environmental | Health impact; Water use/quality |

Table 16. Dictionary mapping of CCLW Objective and Subobjective.

| **Keywords** | **Objective** | **Subobjective** |
| --- | --- | --- |
| 5G | Economic | Technological spillover/innovation |
| Adaptation | |  |
| Afforestation | Environmental | Ecosystem impact |
| Agriculture | Environmental | Ecosystem impact |
| Air Pollution | Social; Environmental | Health impact; Ecosystem impact |
| air pollution | Social; Environmental | Health impact; Ecosystem impact |
| Amazon Forest | Environmental | Ecosystem impact |
| Arctic | Environmental | Ecosystem impact |
| Argentina | |  |
| Aviation |  |  |
| Ban |  |  |
| Banking | Economic |  |
| Biodiversity | Environmental | Biodiversity conservation |
| Biofuels | Environmental; Economic | Ecosystem impact; Energy security |
| Biogas | Social | Energy/mobility access |
| Biomass | Environmental | Ecosystem impact |
| Brt | Social |  |
| Buildings | |  |
| Cap And Trade | |  |
| Car Manufacturing | | |
| Car manufacturing | | |
| Carbon Accounting | | |
| Carbon Budget | |  |
| Carbon Capture And Storage | Environmental | Ecosystem impact |
| Carbon Pricing | |  |
| Carbon Sink | Environmental | Ecosystem impact |
| Ccs | Environmental | Ecosystem impact |
| Cdm | Environmental | Ecosystem impact |
| Central Bank | |  |
| Circular Economy | Economic |  |
| Citizens' Assembly | Social |  |
| Climate Change | |  |
| Climate Change Risks | | |
| Climate Fund | Economic |  |
| Climate Neutrality | | |
| Co Benefits | |  |
| Coal |  |  |
| Coal Mining | Social | Energy/mobility access |
| Coastal Erosion | Environmental | Ecosystem impact |
| Coastal erosion | Environmental | Ecosystem impact |
| Cogeneration | |  |
| Covid 19 | Social | Health impact |
| Covid19 | Social | Health impact |
| Cycling | Environmental | Ecosystem impact |
| Debt For Nature | |  |
| Deforestation | Environmental | Ecosystem impact |
| Digital Transition | Social | Energy/mobility access |
| Disaster Risk Management | Social | Safety/disaster resilience |
| Disclosure | |  |
| Disease Prevention | Social | Health impact |
| Divestment | |  |
| E Vs | Economic | Technological spillover/innovation |
| ETS | Economic |  |
| EV | Economic | Technological spillover/innovation |
| Education | Economic | Technological spillover/innovation |
| Electricity | Economic | Energy security |
| Energy | Economic | Energy security |
| Energy Demand | Social | Energy/mobility access |
| Energy Efficiency | Economic | Productivity/competitiveness |
| Energy Supply | Social | Energy/mobility access |
| Energy storage | Social | Energy/mobility access |
| Environmental Permit | Economic |  |
| European Green Deal | | |
| Ev | Economic | Technological spillover/innovation |
| Finance | Economic |  |
| Fisheries | Environmental | Ecosystem impact |
| Fit |  |  |
| Food Security | Social | Health impact; Food security |
| Forest | Environmental | Ecosystem impact |
| Forests | Environmental | Ecosystem impact |
| Fossil Fuels | Economic | Energy security |
| Fossil Fuels Subsidies | Economic |  |
| Fossil fuels | Economic | Energy security |
| Gas | Economic | Energy security |
| Gender | Social | Gender impact |
| Geothermal | Environmental | Urban heat island effect |
| Germany |  |  |
| Ghg | Environmental | Ecosystem impact |
| Governance | |  |
| Green Bonds | Economic |  |
| Green New Deal | |  |
| Health | Social | Health impact |
| Heat |  |  |
| Human rights | Social |  |
| Hydrogen | Environmental | Ecosystem impact |
| Industry |  |  |
| Infrastructure | |  |
| Institutions / Administrative Arrangements | | |
| Insurance | Economic |  |
| Jobs | Economic | Employment impact |
| Joe Biden | |  |
| Just Transition | |  |
| K-ETS | Economic |  |
| Keystone |  |  |
| Land use | Environmental | Land-use competition |
| Limits on fossil fuels | Economic | Energy security |
| Lulucf | Environmental | Ecosystem impact; Land-use competition |
| Maritime Planning | | |
| Meat | Social | Food security |
| Methane | Environmental | Ecosystem impact |
| Mitigation | |  |
| Mountain | Environmental | Ecosystem impact |
| NAP |  |  |
| National Energy And Climate Plans | | |
| National Security | Social | Safety/disaster resilience |
| Net Zero | Environmental | Ecosystem impact |
| No Adequate Category | | |
| Offshore |  |  |
| Oil | Economic | Energy security |
| Oil and gas | Economic | Energy security |
| Paris Agreement | |  |
| Peat | Economic | Energy security |
| Permit |  |  |
| Physical Activity | Social | Health impact |
| Planning |  |  |
| Public Private | |  |
| Public Transport | |  |
| Pv | Economic | Technological spillover/innovation |
| Redd+ And Lulucf | Environmental | Ecosystem impact; Land-use competition |
| Reforestation | Environmental | Ecosystem impact |
| Reinsurance | Economic |  |
| Renewables | |  |
| Research And Development | Economic | Technological spillover/innovation |
| S Olar | Economic | Technological spillover/innovation |
| SDGs |  |  |
| Shipping |  |  |
| Skills | Economic | Technological spillover/innovation |
| Social Justice | Social |  |
| Solar Panels | Economic | Technological spillover/innovation |
| Spatial Planning | |  |
| Stimulus Plan | Economic |  |
| Stimulus plan | Economic |  |
| Subsidies | Economic |  |
| Tax | Economic |  |
| Tax Incentives | Economic |  |
| Taxes | Economic |  |
| Tourism | Economic |  |
| Trading Scheme | Economic |  |
| Train | Economic | Technological spillover/innovation |
| Transport | |  |
| Transportation | |  |
| Trees | Environmental | Ecosystem impact |
| Unfccc |  |  |
| Walking | Social | Health impact |
| Waste |  |  |
| Water | Social; Environmental | Health impact; Water use/quality |
| Water Management | Environmental | Water use/quality |
| bus |  |  |
| carbon sink | Environmental | Ecosystem impact |
| ccGAP |  |  |
| climate justice | |  |
| coal phase out | |  |
| covid19 | Social | Health impact |
| development | |  |
| energy conservation | Environmental | Ecosystem impact |
| fossil fuel phase out | Economic | Energy security |
| fossil fuels curbing measures | Economic | Energy security |
| infrastructure | |  |
| licensing | |  |
| modal shift | Social | Health impact |
| moratorium | |  |
| truck |  |  |
| Advertising | |  |
| Black carbon | |  |
| CBDR |  |  |
| Cap and Trade | |  |
| Climate protection | Environmental | Ecosystem impact |
| Delta |  |  |
| Development | Economic | New business opportunity / economic activity |
| E-buses |  |  |
| FIT |  |  |
| Food | Social | Food security |
| HFCs |  |  |
| Healthy environment | Social | Health impact |
| Indigenous people | Social |  |
| MRV |  |  |
| Migration | |  |
| Moratorium | Environmental | Biodiversity conservation |
| Nuclear |  |  |
| Nuclear fusion | |  |
| Oceans | Environmental | Biodiversity conservation |
| Procurement | |  |
| Rail |  |  |
| Road |  |  |
| SLCPs |  |  |
| Soil erosion | Environmental | Ecosystem impact |
| Youth |  |  |
| active travel | |  |
| artificialisation | | |
| buses |  |  |
| carbon capture and storage | | |
| carbon credits | |  |
| climate finance | |  |
| co-benefits | |  |
| culture |  |  |
| desertification | Environmental | Ecosystem impact |
| drr |  |  |
| environmental degradation | Environmental | Ecosystem impact |
| equity |  |  |
| flaring |  |  |
| food waste | |  |
| freight |  |  |
| fuels |  |  |
| glaciers | Environmental | Ecosystem impact |
| governance | |  |
| greenwashing | Environmental | Ecosystem impact |
| housing |  |  |
| hydro |  |  |
| hydrofluorocarbons | | |
| innovation | |  |
| intergenerational | | |
| livestock | |  |
| mangroves | Environmental | Ecosystem impact |
| meteorology | |  |
| mining |  |  |
| multi-modal transport | | |
| natural resources | Environmental | Resource / material use impact |
| nitrous oxide | |  |
| off-grid |  |  |
| ozone |  |  |
| power plant | |  |
| rail |  |  |
| rivers | Environmental | Ecosystem impact |
| space |  |  |
| technology | |  |
| wetlands | Environmental | Ecosystem impact |
| wind |  |  |

Table 17. Dictionary mapping of policy titles on Hard law and Soft law.

| **Hard and Soft Law** | **Keywords** |
| --- | --- |
| Constitution | Constitution; Constitutional |
| International Law | Treaties; Treaty |
| Law/Act | Law; Laws; Decree-Law; Bylaw; Act; Acts; Legislation; Legislations; Legislative; Statutes; Statute; Statutory; Ley; LOI; Wet |
| Decree/Order/Ordinance | Ordinance; Ordinances; Order; Orders; Ordice; Decree; Decrees; Decreto; Sub-Decree; Decree-Law; Royal Decree; Royal Decrees; Executive Decree; Executive Decrees; Presidential Decree; Presidential Decrees; Presidential Instruction; Sub-Decree; Real Decreto; Portaria; Ökostromverordnung; DEC; Presidential Instruction |
| Regulation/Directive/Decision | Enforcement Rules; Rules; Rule; Regulations; Regulation; Regulating; Directive; Directives; Decision; Decisions; CFR; Interim Measures; Interim Measure; Interim rules; Interim Procedures; Regulación; Règlement; Despacho Normativo |
| Preparatory Instruments | Green Papers; Green Paper; White Paper; White Papers; Action Programmes; Action Programme; General Programmes; General Programme; Action Plans; Action Plan; Action Program; Action Programs; Incentive Measures; Appropriate Measures; Road Map; Act Programme; Nationally determined contributions; Common Rules; Appropriate Provisions; NDCs; NDC; INDC; Programs; Program; Programme; Programmes; Programming; Milestone; Milestones; Action; Actions; Measures; Arrangements; Arrangement; Prepare; Preparation; Plan; Plans; Planned; Planning; Route; Roadmap; Roadmaps; Blueprint; Agenda; FYP; Scheme; Schemes; Schedules; Schedule; Objectives; Objective; Vision; Pilot; Campaign; Campaigns; Bailout; Goal; Goals; Budget; Targets; Target; Aimed; Aim; Aims; Report; Reporting; Package; Strategy; Strategies; Strategic; Achieving; Achieve; Trials; Tomorrow; Horizon; Bill; Draft; Motion; Mobility; Intention |
| Informative Instruments | Information; Informations; Info; .gov; Website; Survey; Surveys; Progress Report; Report; Reports; Reporting; Evaluation; Assess; Assessment; Consultation; Consult; Consulting; List; Lists; Catalogue; Publicity; Phase; Phase-out; Establish; Established; Found; Set up; Announced; Statement; Reference |
| Interpretative Communications and Notices | Communication; Communications; Interpretation; Interpretative; Interpret; Concept; Explain; Explanation; Explication; Explicate; Exposition; Supplement; Supplements |
| Decisional Notices and Communications | Notification; Notice; Notices; Circulars; Circular; Gazette; Aviso; Communiqué |
| Decisional Guidelines, Codes and Frameworks | Code; Codes; Guidance; Guidances; Guide; Guides; Guideline; Guidelines; Guidelilnes; EnerGuide; Guidebook; Determination; Framework; Frameworks; Outline; Outlines |
| Steering Instruments | Recommendation; Recommendations; Opinion; Opinions; Resolutions; Resolution; Acknowledgements; Acknowledge; Conclusion; Conclusions; ENERGY STAR; Comments; Comment; Declaration; Declarations; Recognitions; Recognition; Recognize; Recognises; Confirmations; Confirmation; Confirm; Desirability; Covenants; Covenant; Debate; Pact; Call; Calls; Partnership; Partner; Partners; Initiative; Initiatives; Advice; Advisory; Proposes; Propose; Proposal; Proposals; Advocates; Advocate; ecoENERGY; Demonstration; Result; Results; Evidence; View; Suggestion; Suggest; Idea; Observation; Say; Calling upon; Inviting; Steering; MOU; MoC; Memorandum; Agreement; Agreements; Protocol; Deal; Commitment; Announcement; Proclamation; Shall; Collaborating; Collaboration; Collaborative; Cooperation; Co-operation |

Table 18. Dictionary mapping of policy contents on Hard law and Soft law.

| **Hard and Soft Law** | **Keywords** |
| --- | --- |
| Constitution | Constitution; Constitutional |
| International Law | Treaties; Treaty |
| Law/Act | Law; Laws; Decree-Law; Bylaw; Act; Acts; Legislation; Legislations; Legislative; Statutes; Statute; Statutory; Ley; LOI; Wet |
| Decree/Order/Ordinance | Ordinance; Ordinances; Order; Orders; Ordice; Decree; Decrees; Decreto; Sub-Decree; Decree-Law; Royal Decree; Royal Decrees; Executive Decree; Executive Decrees; Presidential Decree; Presidential Decrees; Presidential Instruction; Sub-Decree; Real Decreto; Portaria; Ökostromverordnung; DEC; Presidential Instruction |
| Regulation/Directive/Decision | Enforcement Rules; Rules; Rule; Regulations; Regulation; Regulating; Directive; Directives; Decision; Decisions; CFR; Interim Measures; Interim Measure; Interim rules; Interim Procedures; Regulación; Règlement; Despacho Normativo |
| Preparatory Instruments | Green Papers; Green Paper; White Paper; White Papers; Action Programmes; Action Programme; General Programmes; General Programme; Action Plans; Action Plan; Action Program; Action Programs; Incentive Measures; Appropriate Measures; Road Map; Act Programme; Nationally determined contributions; Common Rules; Appropriate Provisions; NDCs; NDC; INDC; Programs; Program; Programme; Programmes; Programming; Milestone; Milestones; Action; Actions; Measures; Arrangements; Arrangement; Activities; Activity; Prepare; Preparation; Plan; Plans; Planned; Planning; Route; Roadmap; Roadmaps; Blueprint; Agenda; FYP; Scheme; Schemes; Schedules; Schedule; Objectives; Objective; Vision; Pilot; Campaign; Campaigns; Bailout; Goal; Goals; Budget; Targets; Target; Aimed; Aim; Aims; Report; Reporting; Package; Strategy; Strategies; Strategic; Achieving; Achieve; Trials; Tomorrow; Horizon; Bill; Motion; Mobility; Intention |
| Informative Instruments | Information; Informations; Info; Website; Survey; Surveys; Progress Report; Report; Reports; Reporting; Evaluation; Assess; Assessment; Consultation; Consult; Consulting; List; Lists; Catalogue; Publicity; Phase; Phase-out; Establish; Established; Found; Set up; Announced; Statement; Reference |
| Interpretative Communications and Notices | Communication; Communications; Interpretation; Interpretative; Interpret; Concept; Explain; Explanation; Explication; Explicate; Exposition; Supplement; Supplements |
| Decisional Notices and Communications | Notification; Notice; Notices; Circulars; Circular; Gazette; Aviso; Communiqué |
| Decisional Guidelines, Codes and Frameworks | Standards; Standard; Standardization; Standardisation; Norm; Norms; Compliance; Aid; Aids; Level; Levels; Label; Labels; Labelling; Rating; Ecolabel; Ecolabels; Eco-label; Taxonomy; Directory; Booklet; Criteria; Tax deductions; Tax deduction; Principles; Principle; Solutions; Solution; NOM; BDS; CP; NCM; NHN; MEPS; MEP; GB; GB/T; LI; LBN; KS; CNS; JS; RS; SANS; SNI; SASO; TIS; VC; NTC; EN; PROCEL; R-2000; AS/NZS; QS; IECC; CBES; HEC; RBES; BEES; NMECC; MUEC; MUBEC; STB; CBECC; ECBC; AS/NZS; GSO; NSO; GOST; NTON; TCVN; SI; S&L; PNS; 50001; UNIT; ISO; Applicable; Performance Certificate; Indexed |
| Steering Instruments | Assistance; Assisted; REN21; Codes of Conduct; Code of Practice; Conduct |

Table 19. Adaptation keywords.

| **Mitigation and Adaptation** | **Keywords** |
| --- | --- |
| Adaption | adaptation; disaster; hazards; desertification; catastrophes; floods; flood; drought; rainwater; erosion; resilience; prevention; emergency; vulnerability; risks; risk; multi-risk; damage; insurance; reef; hydrographic; cold spell; emergency relief; risk management; risk reduction; emergency care; adverse events; disaster management; management of risks; humanitarian crises; disaster preparedness; disaster relief |

Table 20. Entities covered in the training set of GCCMPD.

| Continents | Entities |
| --- | --- |
| North America  (24 Entities) | Antigua and Barbuda; Barbados; Belize; Canada; Costa Rica; Dominican Republic; El Salvador; Guatemala; Honduras; Jamaica; Mexico; Nicaragua; Panama; St. Vincent and the Grenadines; United States; The Bahamas; Cuba; Dominica; Grenada; Greenland; Haiti; St. Kitts and Nevis; St. Luci; Trinidad and Tobago |
| South America  (12 Entities) | Argentina; Venezuela; Brazil; Chile; Colombia; Ecuador; Guyana; Paraguay; Peru; Bolivia; Suriname; Uruguay |
| Asia  (48 Entities) | Armenia; Azerbaijan; Bahrain; Bangladesh; Brunei; China; Cyprus; Georgia; India; Indonesia; Iraq; Iran; Israel; Japan; Jordan; Kazakhstan; Korea; Kuwait; Kyrgyz Republic; Lao PDR; Lebanon; Malaysia; Maldives; Mongolia; Myanmar; Nepal; Oman; Pakistan; Philippines; Qatar; Saudi Arabia; Singapore; Sri Lanka; Tajikistan; Thailand; Turkey; Turkmenistan; United Arab Emirates; Uzbekistan; Vietnam; Afghanistan; Bhutan; Cambodia; Dem. People's Rep. Korea; Syrian Arab Republic; Timor-Leste; Yemen; West Bank and Gaza |
| Europe  (45 Entities) | Albania; Austria; Belarus; Belgium; Bosnia and Herzegovina; Bulgaria; Croatia; Czech Republic; Denmark; Estonia; European Union; Finland; France; Germany; Greece; Hungary; Iceland; Ireland; Italy; Latvia; Lithuania; Luxembourg; Malta; Montenegro; Netherlands; Norway; Poland; Portugal; Moldova; Romania; Russia; Serbia; Slovak Republic; Slovenia; Spain; Sweden; Switzerland; Ukraine; United Kingdom; Andorra; Liechtenstein; Monaco; North Macedonia; San Marino; Kosovo |
| Africa  (53 Entities) | Algeria; Angola; Botswana; Burkina Faso; Burundi; Dem. Rep. Congo; Djibouti; Egypt; Equatorial Guinea; Ethiopia; Gabon; Ghana; Kenya; Lesotho; Libya; Madagascar; Malawi; Mali; Mauritius; Morocco; Mozambique; Namibia; Nigeria; Rwanda; Senegal; Seychelles; South Africa; South Sudan; Tunisia; Uganda; Tanzania; Zambia; Zimbabwe; Benin; Central African Republic; Côte d'Ivoire; Cameroon; Cabo Verde; Eritrea; Guinea; The Gambia; Guinea-Bissau; Liberia; Mauritania; Niger; Sudan; Sierra Leone; Somalia; Sao Tome and Principe; Eswatini; Chad; Togo; Congo |
| Oceania  (17 Entities) | Australia; Fiji; Marshall Islands; Micronesia; Nauru; New Zealand; Samoa; Solomon Islands; Cook Islands; Kiribati; Niue; Palau; Papua New Guinea; French Polynesia; Tonga; Tuvalu; Vanuatu |

Notes: The numbers in the first column represent the count of entities covered in each continent within the training set of GCCMPD.

Table 21. Performance of Instruments Multi-label Classification Model.

| **Model** | **Tradable Allowances** | | | **Regulatory Approaches** | | | **Taxes** | | | **Information Programmes** | | |
| --- | --- | --- | --- | --- | --- | --- | --- | --- | --- | --- | --- | --- |
|  | **Precision** | **Recall** | **F1** | **Precision** | **Recall** | **F1** | **Precision** | **Recall** | **F1** | **Precision** | **Recall** | **F1** |
| LR | 1.00 | 0.21 | 0.35 | 0.76 | 0.66 | 0.70 | 0.36 | 0.05 | 0.10 | 0.81 | 0.57 | 0.67 |
| NB | 0.71 | 0.63 | 0.67 | 0.72 | 0.50 | 0.59 | 0.11 | 0.70 | 0.19 | 0.69 | 0.59 | 0.64 |
| SVM | 1.00 | 0.47 | 0.64 | 0.76 | 0.67 | 0.71 | 0.75 | 0.16 | 0.27 | 0.80 | 0.61 | 0.70 |
| BERT | 1.00 | 0.42 | 0.59 | 0.77 | 0.74 | 0.75 | 0.56 | 0.42 | **0.48** | 0.76 | 0.68 | **0.72** |
| ClimateBERT | 0.78 | 0.74 | **0.76** | 0.78 | 0.76 | **0.77** | 0.49 | 0.47 | **0.48** | 0.70 | 0.70 | 0.70 |
| **Model** | **Government Provision of Public Goods or Services** | | | **Voluntary Actions** | | | **Subsidies** | | | **Macro Average** | | |
|  | **Precision** | **Recall** | **F1** | **Precision** | **Recall** | **F1** | **Precision** | **Recall** | **F1** | **Precision** | **Recall** | **F1** |
| LR | 0.80 | 0.94 | 0.87 | 1.00 | 0.04 | 0.08 | 0.82 | 0.69 | 0.75 | 0.79 | 0.45 | 0.50 |
| NB | 0.75 | 0.89 | 0.82 | 0.28 | 0.46 | 0.35 | 0.71 | 0.54 | 0.61 | 0.57 | 0.62 | 0.55 |
| SVM | 0.82 | 0.94 | **0.88** | 0.88 | 0.14 | 0.24 | 0.80 | 0.75 | 0.77 | 0.83 | 0.54 | 0.60 |
| BERT | 0.85 | 0.89 | 0.87 | 0.74 | 0.34 | 0.47 | 0.81 | 0.78 | **0.79** | 0.78 | 0.61 | 0.67 |
| ClimateBERT | 0.87 | 0.90 | **0.88** | 0.68 | 0.50 | **0.57** | 0.78 | 0.78 | 0.78 | 0.73 | 0.69 | **0.71** |

Table 22. Performance of Sector Multi-label Classification Model.

| **Model** | **Multi-sector** | | | **Energy systems** | | | **Buildings** | | | **AFOLU** | | |
| --- | --- | --- | --- | --- | --- | --- | --- | --- | --- | --- | --- | --- |
|  | **Precision** | **Recall** | **F1** | **Precision** | **Recall** | **F1** | **Precision** | **Recall** | **F1** | **Precision** | **Recall** | **F1** |
| LR | 0.74 | 0.58 | 0.65 | 0.80 | 0.67 | 0.73 | 0.91 | 0.67 | 0.77 | 0.84 | 0.46 | 0.59 |
| NB | 0.43 | 0.87 | 0.57 | 0.43 | 0.88 | 0.58 | 0.69 | 0.75 | 0.72 | 0.11 | 0.85 | 0.20 |
| SVM | 0.73 | 0.62 | 0.67 | 0.80 | 0.73 | 0.76 | 0.91 | 0.75 | 0.82 | 0.85 | 0.60 | 0.70 |
| BERT | 0.71 | 0.75 | 0.73 | 0.76 | 0.83 | **0.79** | 0.85 | 0.79 | 0.82 | 0.67 | 0.81 | **0.73** |
| ClimateBERT | 0.75 | 0.73 | **0.74** | 0.78 | 0.80 | **0.79** | 0.82 | 0.84 | **0.83** | 0.66 | 0.79 | 0.72 |
| **Model** | **Transport** | | | **Industry** | | | **Macro Average** | | |  |  |  |
|  | **Precision** | **Recall** | **F1** | **Precision** | **Recall** | **F1** | **Precision** | **Recall** | **F1** |  |  |  |
| LR | 0.92 | 0.60 | 0.73 | 0.63 | 0.19 | 0.30 | 0.81 | 0.53 | 0.63 |  |  |  |
| NB | 0.38 | 0.79 | 0.52 | 0.16 | 0.77 | 0.27 | 0.37 | 0.82 | 0.48 |  |  |  |
| SVM | 0.95 | 0.67 | 0.79 | 0.70 | 0.31 | 0.43 | 0.82 | 0.61 | 0.70 |  |  |  |
| BERT | 0.82 | 0.81 | 0.82 | 0.51 | 0.60 | 0.55 | 0.72 | 0.77 | 0.74 |  |  |  |
| ClimateBERT | 0.90 | 0.80 | **0.84** | 0.58 | 0.56 | **0.57** | 0.75 | 0.75 | **0.75** |  |  |  |

Table 23. Performance of Objective Multi-label Classification Model.

| **Model** | **Environmental** | | | **Social** | | | **Economic** | | | **Macro Average** | | |
| --- | --- | --- | --- | --- | --- | --- | --- | --- | --- | --- | --- | --- |
|  | **Precision** | **Recall** | **F1** | **Precision** | **Recall** | **F1** | **Precision** | **Recall** | **F1** | **Precision** | **Recall** | **F1** |
| LR | 0.81 | 0.43 | 0.56 | 0.72 | 0.34 | 0.46 | 0.82 | 0.75 | 0.78 | 0.78 | 0.51 | 0.60 |
| NB | 0.20 | 0.82 | 0.32 | 0.21 | 0.82 | 0.34 | 0.74 | 0.51 | 0.61 | 0.38 | 0.72 | 0.42 |
| SVM | 0.83 | 0.56 | 0.66 | 0.75 | 0.42 | 0.54 | 0.82 | 0.78 | 0.80 | 0.80 | 0.59 | 0.67 |
| BERT | 0.76 | 0.68 | 0.71 | 0.69 | 0.68 | **0.69** | 0.87 | 0.83 | **0.85** | 0.77 | 0.73 | **0.75** |
| ClimateBERT | 0.74 | 0.71 | **0.73** | 0.68 | 0.67 | 0.68 | 0.85 | 0.84 | 0.84 | 0.75 | 0.74 | **0.75** |

Table 24. Performance of Binding force Single-label Classification Model.

| **Model** | **Law/Act** | | | **Decree/Order/Ordinance** | | | **Regulation/Directive/Decision** | | | **Preparatory Instruments** | | |
| --- | --- | --- | --- | --- | --- | --- | --- | --- | --- | --- | --- | --- |
|  | **Precision** | **Recall** | **F1** | **Precision** | **Recall** | **F1** | **Precision** | **Recall** | **F1** | **Precision** | **Recall** | **F1** |
| LR | 0.80 | 0.83 | 0.81 | 0.96 | 0.51 | 0.66 | 0.74 | 0.59 | 0.65 | 0.67 | 0.96 | 0.79 |
| NB | 0.23 | 0.90 | 0.37 | 0.70 | 0.23 | 0.35 | 0.67 | 0.28 | 0.40 | 0.78 | 0.29 | 0.42 |
| SVM | 0.77 | 0.88 | 0.82 | 0.79 | 0.64 | 0.70 | 0.68 | 0.69 | 0.68 | 0.74 | 0.93 | 0.82 |
| BERT | 0.93 | 0.95 | **0.94** | 0.90 | 0.90 | **0.90** | 0.88 | 0.94 | **0.91** | 0.87 | 0.89 | **0.88** |
| ClimateBERT | 0.92 | 0.94 | 0.93 | 0.88 | 0.86 | 0.87 | 0.80 | 0.92 | 0.85 | 0.87 | 0.89 | **0.88** |
| **Model** | **Informative Instruments** | | | **Decisional Notices and Communications** | | | **Interpretative Communications and Notices** | | | **Decisional Guidelines, Codes and Frameworks** | | |
|  | **Precision** | **Recall** | **F1** | **Precision** | **Recall** | **F1** | **Precision** | **Recall** | **F1** | **Precision** | **Recall** | **F1** |
| LR | 1.00 | 0.00 | 0.00 | 1.00 | 0.17 | 0.29 | 1.00 | 0.00 | 0.00 | 0.71 | 0.56 | 0.63 |
| NB | 0.09 | 0.11 | 0.10 | 0.67 | 0.67 | 0.67 | 1.00 | 0.25 | 0.40 | 0.74 | 0.26 | 0.39 |
| SVM | 0.50 | 0.05 | 0.10 | 0.71 | 0.83 | 0.77 | 1.00 | 0.00 | 0.00 | 0.75 | 0.55 | 0.64 |
| BERT | 0.49 | 0.55 | **0.52** | 0.60 | 1.00 | 0.75 | 1.00 | 0.50 | 0.67 | 0.81 | 0.70 | **0.75** |
| ClimateBERT | 0.50 | 0.39 | 0.44 | 0.67 | 1.00 | **0.80** | 0.75 | 0.75 | **0.75** | 0.80 | 0.70 | 0.74 |
| **Model** | **Steering Instruments** | | | **Other Strategy Plan or Target** | | | **Macro Average** | | |  |  |  |
|  | **Precision** | **Recall** | **F1** | **Precision** | **Recall** | **F1** | **Precision** | **Recall** | **F1** |  |  |  |
| LR | 0.67 | 0.33 | 0.44 | 0.56 | 0.35 | 0.43 | 0.81 | 0.43 | 0.47 |  |  |  |
| NB | 0.48 | 0.13 | 0.21 | 0.19 | 0.19 | 0.19 | 0.56 | 0.33 | 0.35 |  |  |  |
| SVM | 0.72 | 0.42 | 0.53 | 0.58 | 0.40 | 0.48 | 0.72 | 0.54 | 0.55 |  |  |  |
| BERT | 0.71 | 0.68 | **0.69** | 0.55 | 0.58 | 0.56 | 0.77 | 0.77 | **0.76** |  |  |  |
| ClimateBERT | 0.66 | 0.62 | 0.64 | 0.57 | 0.60 | **0.58** | 0.74 | 0.77 | 0.75 |  |  |  |

Table 25. Performance of Executive/legislative Single-label Classification Model.

| **Model** | **Legislative** | | | **Executive** | | | **Macro Average** | | |
| --- | --- | --- | --- | --- | --- | --- | --- | --- | --- |
|  | **Precision** | **Recall** | **F1** | **Precision** | **Recall** | **F1** | **Precision** | **Recall** | **F1** |
| LR | 0.91 | 0.70 | 0.79 | 0.95 | 0.99 | 0.97 | 0.93 | 0.85 | 0.88 |
| NB | 0.23 | 0.61 | 0.34 | 0.91 | 0.66 | 0.77 | 0.57 | 0.64 | 0.55 |
| SVM | 0.89 | 0.77 | 0.83 | 0.96 | 0.98 | 0.97 | 0.93 | 0.88 | 0.90 |
| BERT | 0.99 | 0.97 | **0.98** | 0.99 | 1.00 | **1.00** | 0.99 | 0.98 | **0.99** |
| ClimateBERT | 0.99 | 0.96 | 0.97 | 0.99 | 1.00 | **1.00** | 0.99 | 0.98 | **0.99** |

Table 26. Criteria for judging jurisdictions based on named entity recognition results.

| **Jurisdiction** | **Specific basis for judgment** |
| --- | --- |
| International | Identify two or more different countries |
| Subnational region | Identify two or more different states/provinces |
| SubNational | Identify one state/province |
| National | Identify one country |

Note: The priority when the judgment criteria are met at the same time is from top to bottom.

Table 27. Key Results Documents from GCCMPD.

| **Files** | **Description** |
| --- | --- |
| **Policy** | |
| *iea_cp_cclw.xlsx* | Manual inspection and verification policy as of December 31, 2021 |
| *iea_cp_cclw_update.xlsx* | The merged results of the updated IEA, CP, and CCLW datasets (the parts that have been manually checked are retained through matching) |
| *all_policies_adaptation_result.xlsx* | Policies for climate adaptation (with allocation of multilateral and bilateral policies) |
| *all_policies_adaptation_result_for_topic.xlsx* | Policies for climate adaptation (without allocation of multilateral and bilateral policies) |
| *all_policies_mitigation_result.xlsx* | Policies for climate mitigation (with allocation of multilateral and bilateral policies) |
| *all_policies_mitigation_result_for_topic.xlsx* | Policies for climate mitigation (without allocation of multilateral and bilateral policies) |
| *bm25_move_duplicate_result.xlsx* | Optimal BM25 value, duplicate policy information, original version (with allocation of multilateral and bilateral policies) |
| *bm25_move_duplicate_result_for_topic.xlsx* | Optimal BM25 value, duplicate policy information, original version (without allocation of multilateral and bilateral policies) |
| **Topic** | |
| *Topic_country_expand.xlsx* | Topic model based on *all_policies_mitigation_result.xlsx* |
| *Topic_country_expand_hierarchical_topics.xlsx* | Hierarchical Topic Model based on *all_policies_mitigation_result.xlsx* |
| *Topic_country_expand_topics_over_time.xlsx* | Dynamic Topic Model based on *all_policies_mitigation_result.xlsx* |
| *Topic_except_ecolex.xlsx* | Topic model based on *all_policies_mitigation_result.xlsx* after excluding ECOLEX sources |
| *Topic_except_ecolex_topics_over_time.xlsx* | Dynamic Topic Model based on *all_policies_mitigation_result.xlsx* after excluding ECOLEX sources |
| *Topic_hierarchical_topics_except_ecolex.xlsx* | Hierarchical Topic Model based on *all_policies_mitigation_result.xlsx* after excluding ECOLEX sources |
| *Topic_iea_cp_cclw.xlsx* | Topic model based on *iea_cp_cclw.xlsx* |
| *Topic_iea_cp_cclw_hierarchical_topics.xlsx* | Hierarchical Topic Model based on *iea_cp_cclw.xlsx* |
| *Topic_iea_cp_cclw_topics_over_time.xlsx* | Dynamic Topic Model based on *iea_cp_cclw.xlsx* |
| *Topic.xlsx* | Topic model based on *all_policies_mitigation_result_for_topic.xlsx* |
| *Topic_topics_over_time.xlsx* | Dynamic Topic Model based on *all_policies_mitigation_result_for_topic.xlsx* |
| *Topic_hierarchical_topics.xlsx* | Hierarchical Topic Model based on *all_policies_mitigation_result_for_topic.xlsx* |

Table 28. Comparison of dictionary mapping and manual checking results on Sector-Instrument.

| **Sector-Instrument** | **Dictionary Mapping vs Manual check** | | | |
| --- | --- | --- | --- | --- |
|  | **Precision** | **Recall** | **F1** | **N** |
| **Government Provision of Public Goods or Services** | | | | |
| Brokerage for industrial cooperation | 0.29 | 1.00 | 0.44 | 4 |
| Infrastructure expansion (district heating / cooling or common carrier) | 0.90 | 0.96 | 0.93 | 102 |
| Investment in alternative fuel infrastructure | 0.88 | 0.99 | 0.93 | 104 |
| Investment in improvement and diffusion of innovative technologies in agriculture and forestry | 1.00 | 1.00 | 1.00 | 204 |
| Investment in transit and human powered transport | 0.00 | 1.00 | 0.00 | 0 |
| Low emission vehicle procurement | 0.91 | 0.98 | 0.94 | 40 |
| Protection of national, state, and local forests | 1.00 | 1.00 | 1.00 | 57 |
| Public procurement of efficient buildings and appliances | 0.97 | 0.89 | 0.93 | 72 |
| Research and development | 0.96 | 0.96 | 0.96 | 459 |
| Training and education | 0.99 | 0.96 | 0.98 | 142 |
| **Information Programmes** | | | | |
| Benchmarking | 0.00 | 1.00 | 0.00 | 0 |
| Brokerage for industrial cooperation | 0.00 | 1.00 | 0.00 | 0 |
| Certification schemes for sustainable forest practices | 0.00 | 1.00 | 0.00 | 0 |
| Energy advice programmes | 0.97 | 0.93 | 0.95 | 138 |
| Energy audits | 0.98 | 0.95 | 0.97 | 465 |
| Fuel labelling | 0.99 | 0.94 | 0.96 | 145 |
| Information policies to support REDD+ including monitoring, reporting and verification | 1.00 | 1.00 | 1.00 | 74 |
| Labelling programmes | 1.00 | 1.00 | 1.00 | 263 |
| Vehicle efficiency labelling | 0.83 | 1.00 | 0.91 | 5 |
| **Regulatory Approaches** | | | | |
| Air and water pollution control GHG precursors | 1.00 | 1.00 | 1.00 | 75 |
| Building codes and standards | 0.96 | 0.88 | 0.92 | 448 |
| Efficiency or environmental performance standards | 0.98 | 0.94 | 0.96 | 348 |
| Energy efficiency standards for equipment | 0.97 | 0.87 | 0.91 | 308 |
| Energy management systems | 0.56 | 1.00 | 0.71 | 5 |
| Equipment and appliance standards | 0.98 | 0.99 | 0.99 | 506 |
| Equitable access to electricity grid | 0.78 | 1.00 | 0.88 | 14 |
| Forest law to reduce deforestation | 0.00 | 1.00 | 0.00 | 0 |
| Fuel economy performance standards | 0.74 | 0.97 | 0.84 | 115 |
| Fuel quality standards | 0.75 | 0.53 | 0.62 | 57 |
| GHG emission performance standards | 1.00 | 0.95 | 0.97 | 219 |
| Labelling and public procurement regulations | 0.00 | 1.00 | 0.00 | 0 |
| Land-use planning and governance | 0.00 | 1.00 | 0.00 | 0 |
| Legal status of longterm CO2 storage | 0.00 | 1.00 | 0.00 | 0 |
| Mandates for energy retailers to assist customers invest in energy efficiency | 1.00 | 0.86 | 0.92 | 21 |
| National policies to support REDD+ including monitoring, reporting and verification | 0.00 | 1.00 | 0.00 | 0 |
| Regulatory restrictions to encourage modal shifts (road to rail) | 0.00 | 1.00 | 0.00 | 0 |
| Renewable Portfolio Standards for renewable energy | 0.79 | 0.90 | 0.84 | 63 |
| Restriction on use of vehicles in certain areas | 0.00 | 1.00 | 0.00 | 0 |
| Urban planning and zoning restrictions | 0.00 | 1.00 | 0.00 | 0 |
| Voluntary agreements (where bound by regulation) | 0.00 | 1.00 | 0.00 | 0 |
| **Subsidies** | | | | |
| Biofuel subsidies | 0.00 | 1.00 | 0.00 | 0 |
| Capital subsidies and insurance for 1st generation Carbon Dioxide Capture and Storage (CCS) | 0.25 | 0.67 | 0.36 | 3 |
| Credit lines for low carbon agriculture, sustainable forestry | 0.90 | 1.00 | 0.95 | 9 |
| Feebates | 0.60 | 1.00 | 0.75 | 6 |
| Feed-in-tariffs for renewable energy | 0.82 | 1.00 | 0.90 | 232 |
| Fiscal incentives | 0.82 | 0.92 | 0.87 | 97 |
| Fossil fuel subsidy removal | 0.50 | 1.00 | 0.67 | 1 |
| Subsidies | 0.00 | 1.00 | 0.00 | 0 |
| Subsidies or tax exemptions for investment in efficient buildings, retrofits and products | 0.56 | 0.77 | 0.65 | 53 |
| Subsidized loans | 0.93 | 0.94 | 0.93 | 81 |
| Vehicle purchase subsidies | 0.00 | 1.00 | 0.00 | 0 |
| **Taxes** | | | | |
| Carbon and/or energy taxes | 0.55 | 0.73 | 0.63 | 15 |
| Carbon tax or energy tax | 0.83 | 0.76 | 0.79 | 33 |
| Carbon taxes | 0.52 | 0.76 | 0.62 | 33 |
| Congestion charges, vehicle registration fees, road tolls | 0.00 | 1.00 | 0.00 | 0 |
| Fertilizer or Nitrogen taxes to reduce nitrous oxide | 0.80 | 0.73 | 0.76 | 11 |
| Fuel taxes | 0.66 | 0.92 | 0.77 | 60 |
| Vehicle taxes | 0.00 | 1.00 | 0.00 | 0 |
| Waste disposal taxes or charges | 0.00 | 1.00 | 0.00 | 0 |
| **Tradable Allowances** | | | | |
| Compliance schemes outside Kyoto protocol (national schemes) | 0.00 | 1.00 | 0.00 | 0 |
| Emission credits under CDM | 0.33 | 0.50 | 0.40 | 2 |
| Emission credits under the Kyoto Protocol's Clean Development Mechanism (CDM) | 0.00 | 1.00 | 0.00 | 0 |
| Emissions trading | 0.77 | 1.00 | 0.87 | 33 |
| Fuel and vehicle standards | 0.86 | 0.86 | 0.86 | 7 |
| Tradable certificates for energy efficiency improvements (white certificates) | 0.67 | 0.57 | 0.62 | 7 |
| Tradable Green Certificates | 0.38 | 1.00 | 0.55 | 3 |
| **Voluntary Actions** | | | | |
| Labelling programmes for efficient buildings | 0.00 | 1.00 | 0.00 | 0 |
| Product eco-labelling | 0.00 | 1.00 | 0.00 | 0 |
| Voluntary agreements on energy targets or adoption of energy management systems, or resource efficiency | 0.25 | 1.00 | 0.40 | 2 |
|  | | | | |
| Micro Average | 0.88 | 0.94 | 0.91 | 5,141 |
| Macro Average | 0.52 | 0.94 | 0.55 | 5,141 |
| Weighted Average | 0.93 | 0.94 | 0.93 | 5,141 |
| Samples Average | 0.96 | 0.98 | 0.95 | 5,141 |

Table 29. Comparison of dictionary mapping and manual checking results on Subsector.

| **Subsector** | **Dictionary Mapping vs Manual check** | | | |
| --- | --- | --- | --- | --- |
|  | **Precision** | **Recall** | **F1** | **N** |
| **AFOLU** | | | | |
| Managed soils and pasture | 0.00 | 1.00 | 0.00 | 0 |
| Manure management | 0.00 | 1.00 | 0.00 | 0 |
| Rice cultivation | 0.00 | 1.00 | 0.00 | 0 |
| **Buildings** | | | | |
| Non-residential | 0.96 | 0.66 | 0.78 | 822 |
| Residential | 0.95 | 0.76 | 0.84 | 1,148 |
| **Energy systems** | | | | |
| Biomass energy systems | 0.00 | 1.00 | 0.00 | 0 |
| Coal mining fugitive emissions | 0.77 | 1.00 | 0.87 | 10 |
| Electricity & heat | 0.95 | 0.96 | 0.95 | 2,149 |
| Oil and gas fugitive emissions | 0.96 | 0.96 | 0.96 | 254 |
| Petroleum refining | 0.67 | 1.00 | 0.80 | 6 |
| **Industry** | | | | |
| Cement | 0.00 | 1.00 | 0.00 | 0 |
| Chemicals | 0.30 | 0.91 | 0.45 | 11 |
| Metals | 0.31 | 1.00 | 0.47 | 8 |
| Other industry | 0.46 | 0.93 | 0.62 | 61 |
| Waste | 0.93 | 0.97 | 0.95 | 187 |
| **Transport** | | | | |
| Domestic Aviation | 0.83 | 1.00 | 0.91 | 50 |
| Inland Shipping | 0.83 | 1.00 | 0.91 | 53 |
| International Aviation | 0.29 | 1.00 | 0.44 | 2 |
| International Shipping | 0.33 | 1.00 | 0.50 | 1 |
| Rail | 0.64 | 1.00 | 0.78 | 51 |
| Road | 0.93 | 1.00 | 0.96 | 841 |
|  | | | | |
| Micro Average | 0.91 | 0.88 | 0.90 | 5,654 |
| Macro Average | 0.53 | 0.96 | 0.58 | 5,654 |
| Weighted Average | 0.93 | 0.88 | 0.90 | 5,654 |
| Samples Average | 0.97 | 0.97 | 0.94 | 5,654 |

Table 30. Comparison of dictionary mapping and manual checking results on Subobjective.

| **Subobjective** | **Dictionary Mapping vs Manual check** | | | |
| --- | --- | --- | --- | --- |
|  | **Precision** | **Recall** | **F1** | **N** |
| **Economic** | | | | |
| Employment impact | 0.06 | 1.00 | 0.12 | 7 |
| Energy security | 0.70 | 1.00 | 0.82 | 210 |
| New business opportunity/economic activity | 0.50 | 1.00 | 0.67 | 1 |
| Productivity/competitiveness | 0.99 | 0.99 | 0.99 | 3,399 |
| Technological spillover/innovation | 0.97 | 0.99 | 0.98 | 946 |
| **Environmental** | | | | |
| Biodiversity conservation | 0.14 | 1.00 | 0.24 | 28 |
| Ecosystem impact | 0.82 | 0.99 | 0.90 | 937 |
| Land-use competition | 0.84 | 1.00 | 0.91 | 285 |
| Resource/material use impact | 0.36 | 1.00 | 0.53 | 10 |
| Urban heat island effect | 0.80 | 1.00 | 0.89 | 4 |
| Water use/quality | 0.29 | 1.00 | 0.45 | 31 |
| **Social** | | | | |
| (Fuel) Poverty alleviation | 0.44 | 1.00 | 0.62 | 16 |
| Energy/mobility access | 0.96 | 0.99 | 0.98 | 1,322 |
| Food security | 0.27 | 1.00 | 0.42 | 31 |
| Gender impact | 0.33 | 1.00 | 0.50 | 20 |
| Health impact | 0.47 | 1.00 | 0.64 | 251 |
| Safety/disaster resilience | 0.21 | 1.00 | 0.35 | 7 |
|  | | | | |
| Micro Average | 0.85 | 0.99 | 0.92 | 7,505 |
| Macro Average | 0.54 | 1.00 | 0.65 | 7,505 |
| Weighted Average | 0.91 | 0.99 | 0.94 | 7,505 |
| Samples Average | 0.94 | 1.00 | 0.95 | 7,505 |

1. Sub-sector, sector-instrument and sub-objective cannot be classified in the data expansion part of the dataset because the classification is too sparse. [↑](#footnote-ref-1)
2. Best Match 25 algorithm, a ranking algorithm based on probabilistic relevance framework for information retrieval and search engines, can generate the similarity score between two policies. It enhances the traditional TFIDF (Term Frequency-Inverse Document Frequency) model. [↑](#footnote-ref-2)
3. Term Frequency-Inverse Document Frequency [↑](#footnote-ref-3)
4. Principal component analysis [↑](#footnote-ref-4)
5. Usually, for categories with a small sample size, the performance of the model is low, but the weight of the model evaluation is equal to that of categories with a large sample size. [↑](#footnote-ref-5)
6. Country and year are controlled by grouping, but city information (e.g., Victoria and Queensland) is still what separates the two policies. [↑](#footnote-ref-6)
7. The reason for using the optimal rank instead of the optimal score is that although the BM25 algorithm has optimized the number and length of documents, the same two texts will still have different similarity scores in different corpora (usually the similarity score increases when the corpus become larger). [↑](#footnote-ref-7)
8. As of the author's writing, the latest version of the CCLW incorporates Capacity Building into Governance, expands Incentives and changes it to Economic, and adds Information: Direct Investment, Economic, Governance, Information, Regulation. [↑](#footnote-ref-8)
9. Note that the CCLW differs from Dubash et al. (2013) and Iacobuta et al. (2018) in this category, which covers some goals, such as China's Five year plan, which was approved by People's Congress and is already in force, and therefore it is categorized as national climate legislation, while CCLW treats it as an executive order from the perspective of the legislature. [↑](#footnote-ref-9)
